# Supplementary material for: Variants in EXOSC9 Disrupt the RNA Exosome and Result in Cerebellar Atrophy with Spinal Motor Neuronopathy
Source: Am J Hum Genet. 2018 May 3;102(5):858–73. doi: 10.1016/j.ajhg.2018.03.011 (PMC5986733; doi:10.1016/j.ajhg.2018.03.011)
Supplement: Document S1. Figure S1 and Tables S1–S3 [file mmc1.pdf]

The American Journal of Human Genetics, Volume *102*

Supplemental Data

Variants in *EXOSC9* Disrupt the RNA Exosome and Result in Cerebellar Atrophy with Spinal Motor Neuronopathy

David T. Burns, Sandra Donkervoort, Juliane S. Müller, Ellen Knierim, Diana Bharucha-Goebel, Eissa Ali Faqeih, Stephanie K. Bell, Abdullah Y. AlFaifi, Dorota Monies, Francisca Millan, Kyle Retterer, Sarah Dyack, Sara MacKay, Susanne Morales-Gonzalez, Michele Giunta, Benjamin Munro, Gavin Hudson, Mena Scavina, Laura Baker, Tara C. Massini, Monkol Lek, Ying Hu, Daniel Ezzo, Fowzan S. AlKuraya, Peter B. Kang, Helen Griffin, A. Reghan Foley, Markus Schuelke, Rita Horvath, and Carsten G. Bönnemann

Figure S1:

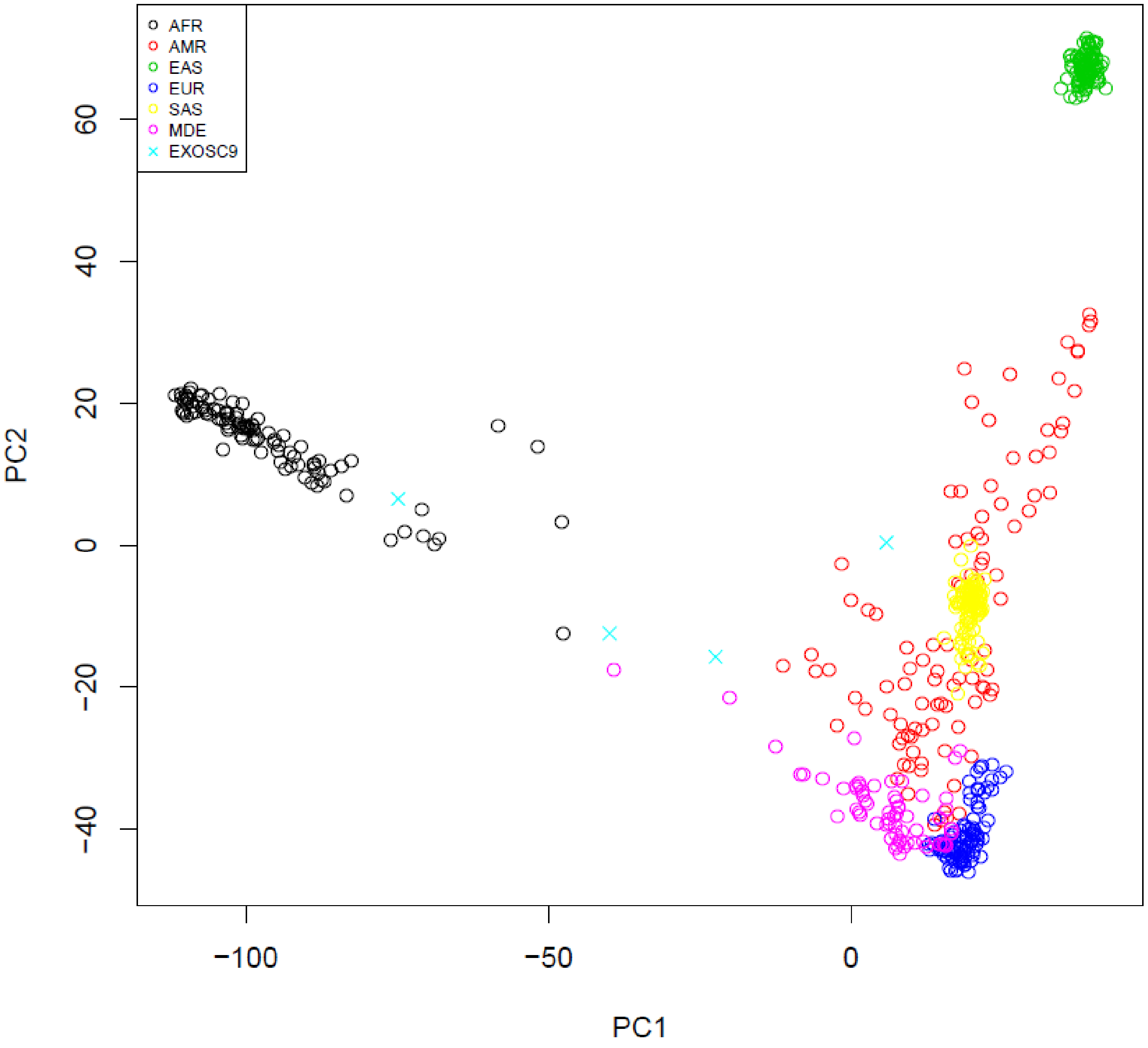

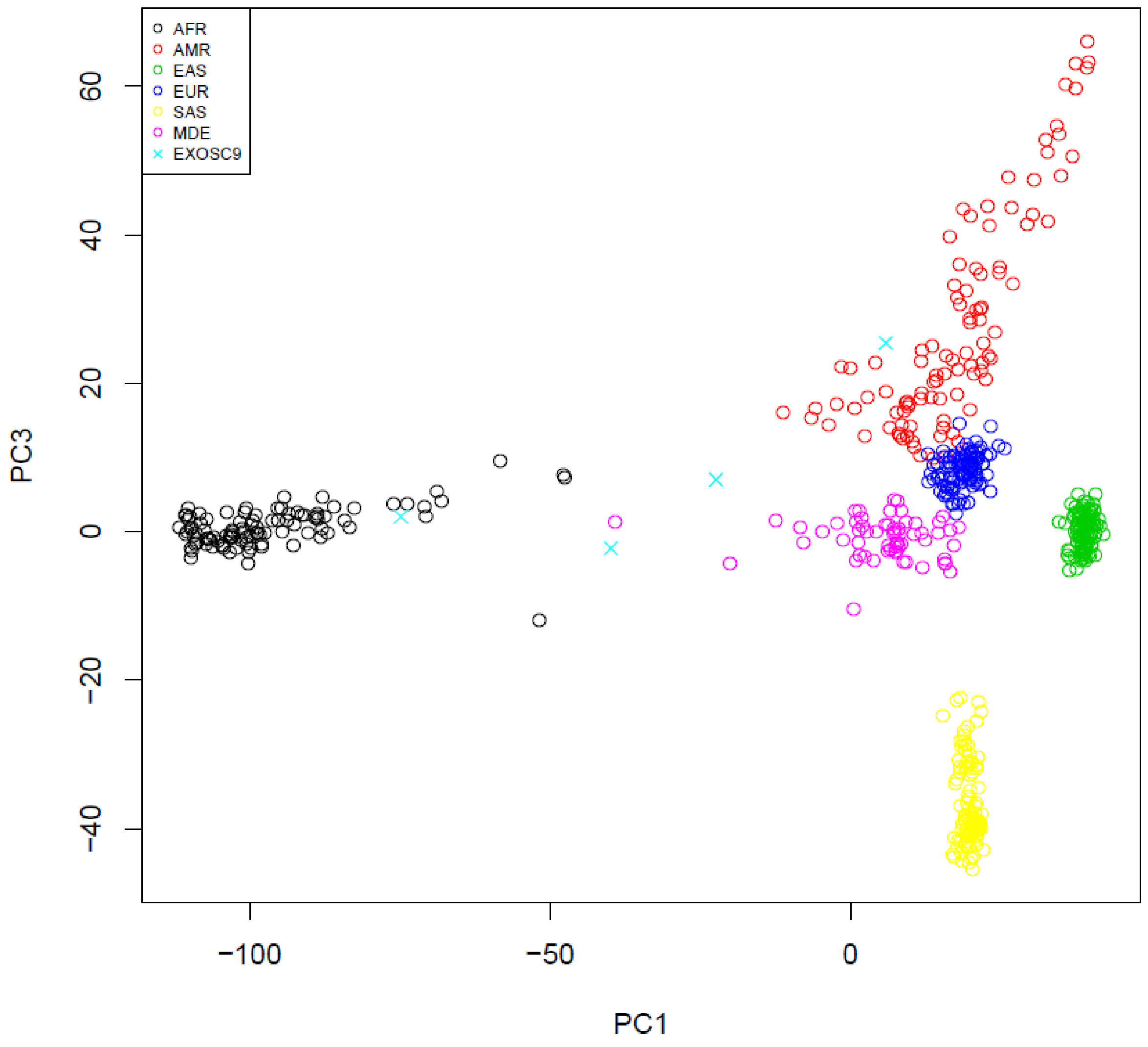
AFR
AMR
EAS
EUR
SAS
MDE
EXOSC9
PC3
60
40
20
0
-20
-40
-100
-50
0
PC1

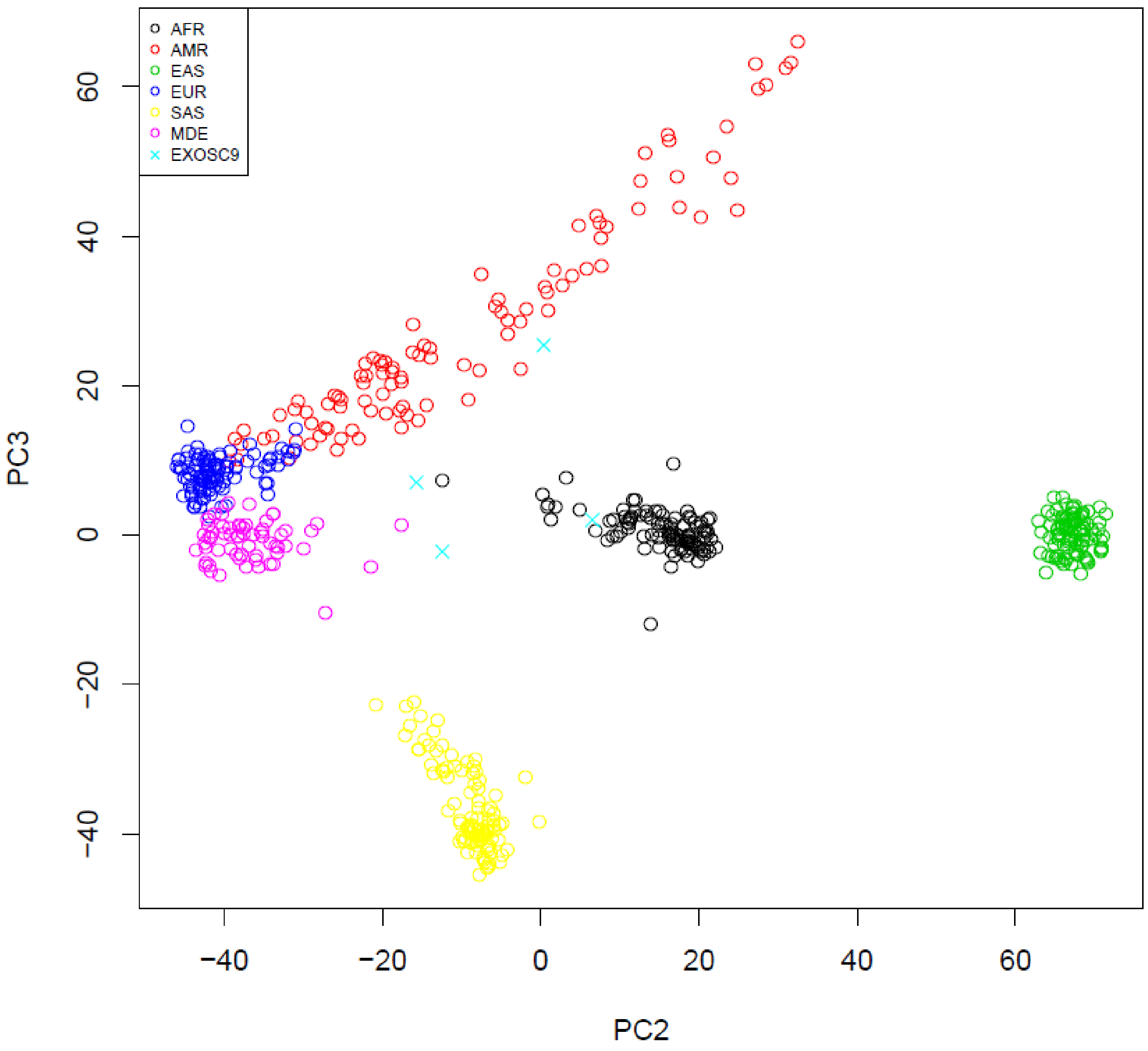
AFR
AMR
EAS
EUR
SAS
MDE
EXOSC9
PC3
PC2
60
40
20
0
−20
−40
−40
−20
0
20
40
60

Figure S1: Principal Component Analysis of exome SNP genotypes compared against random representative samples from the 1000 Genomes Projects Phase 3 super-populations plus a GeneDx-sequenced Middle Eastern (MDE) population. Five total components were calculated (see Table) but only the three most informative components are shown.

Table S1:

Primer name	Forward 3´-5´	Reverse 3´-5´
ZF *exosc9* (I1E2 MO)	CAAACTGCGAGAGGCTTTTCC	TCTCCTGAAACTACACAGAGAG
ZF *exosc9* (E3 gRNA)	TGAGTCAGGTATCCTGTGAGC	TGCAAGAATGTTAACCTGTTTGGTT
pUC/M13	CGCCAGGGTTTTCCCAGTCACGAC	TCACACAGGAAACAGCTATGAC
**Morpholino**	**Sequence**	
ZF *exosc9* morpholino	GCGCTACAAAACGGTACACAGATAA	

Table S1: Primer sequences used in zebrafish

Table S2:

	Characteristics and Symptoms	HPO ID	Individual 1	Individual 2	Individual 3	Individual 4	Percentage
Mutation in EXOSC9			homo c.41T>C	comp het c.41T>C | c.481C>T	homo c.41T>C	homo c.41T>C	
Effect on translation			p.(Leu14Pro)	p.(Leu14Pro), p.(Arg161*)	p.(Leu14Pro)	p.(Leu14Pro)	
Origin			El Salvador	African-Canadian-Jamaican	Saudi Arabia	African-European-Filippino	
Gender			female	male	female	female	
Age at onset			8 mo	birth	birth	2 weeks	
Age at last assessment			28 mo		4.5 y	19 mo	
Age at death				15 mo			
**Head**							
	Microcephalus	HP:0000252			+		25
	Low-set ears	HP:0000369		+			25
	Short neck	HP:0000470		+			25
	Blue sclerae	HP:0000592		+			25
	Prominent epicanthic folds	HP:0007930		+			25
	Hypertelorism	HP:0000316		+			25
**Respiratory and chest**							
	Recurrent infections	HP:0002719	+				25
	Respiratory failure in infancy	HP:0002878	+	+			50
**Abdomen**							
	Gastrostomy tube feeding in infancy	HP:0011471	+	+			50
	Gastrooesophageal reflux	HP:0002020	+				25
	Feeding difficulties in infancy	HP:0008872			+		25
**Skeletal**							
	Arthrogryposis multiplex congenital	HP:0002804	+	+			50
	Congenital fractures	HP:0005855		+			25
**Neurologic**							
	Severe muscular hypotonia	HP:0006829	+	+	+	+	**100**
	Seizures	HP:0001250			+		25
	Type I muscle fiber predominance	HP:0003803	+	+		+	**75**
	Poor head control	HP:0002421	+			+	50
	Failure to thrive	HP:0001508		+			25
	Generalized muscle weakness	HP:0003324	+		+	+	**75**
	Nystagmus	HP: 0000639	+			+	50
	Cerebellar atrophy	HP:0001272	+	+		+	75
	Vermis atrophy	HP:0001284	+				25
	Cerebral atrophy	HP:0002059		+	+		50
	Areflexia	HP:0001284	+				25
	Increased muscle reflexes	HP:0001347			+		25
	EMG: neuropathic changes	HP:0003445	+			+	50
	Muscle / tongue fasciculations	HP:0002380	+				25
	Delayed gross motor development	HP:0002194	+	+	+	+	**100**
	Weak voice	HP:0001621	+		+	+	**75**
**Prenatal manifestation**							
	Decreased fetal movements	HP:0001558	+	+			30
	Oligohydramnios	HP:0001562		+			25
	Intrauterine growth retardation (< 10th percentile)	HP:0001511		+			25

Table S2: HPO coding of phenotypes due to mutations in *EXOSC9*

Table S3:

Sample	knn.pred	PC1	PC2	PC3	PC4	PC5
**Individual 1**	AMR	5.86084161991711	0.381779639737347	25.4032514365166	-21.0875664424482	18.6590532074565
**Individual 2**	AMR	-22.3702289478425	-15.7062479478535	7.02632626688231	13.8203817951703	34.1568567434397
**Individual 3**	AFR	-39.8713917778595	-12.421418841825	-2.22439179095596	17.719501768131	39.8300579056514
**Individual 4**	AFR	-74.7967964546837	6.5332515180803	2.02902721367773	3.38496964411094	3.05927032223124

Table S3: Principal Component Analysis of exome SNP genotypes compared against random representative samples from the 1000 Genomes Projects Phase 3 major populations plus a GeneDx-sequenced Middle Eastern (MDE) population. Components are numbered in the order of informativeness and knn.pred is the nearest-neighbor-predicted population.
