# Supplementary material for: Variants in EXOSC9 Disrupt the RNA Exosome and Result in Cerebellar Atrophy with Spinal Motor Neuronopathy
Source: Am J Hum Genet. 2018 May 3;102(5):858–73. doi: 10.1016/j.ajhg.2018.03.011 (PMC5986733; doi:10.1016/j.ajhg.2018.03.011)
Supplement: Document S2. Article plus Supplemental Data [file mmc4.pdf]

ARTICLE

Variants in *EXOSC9* Disrupt the RNA Exosome and Result in Cerebellar Atrophy with Spinal Motor Neuronopathy

David T. Burns,[1,15] Sandra Donkervoort,[2,15] Juliane S. Müller,[1,15] Ellen Knierim,[3,15] Diana Bharucha-Goebel,[2,4] Eissa Ali Faqeih,[5] Stephanie K. Bell,[6] Abdullah Y. AlFaifi,[5] Dorota Monies,[7] Francisca Millan,[8] Kyle Retterer,[8] Sarah Dyack,[9] Sara MacKay,[10] Susanne Morales-Gonzalez,[3] Michele Giunta,[1] Benjamin Munro,[1] Gavin Hudson,[1] Mena Scavina,[11] Laura Baker,[12] Tara C. Massini,[13] Monkol Lek,[14] Ying Hu,[2] Daniel Ezzo,[2] Fowzan S. AlKuraya,[7] Peter B. Kang,[6] Helen Griffin,[1] A. Reghan Foley,[2] Markus Schuelke,[3,16] Rita Horvath,[1,16,*] and Carsten G. Bönnemann[2,16]

The exosome is a conserved multi-protein complex that is essential for correct RNA processing. Recessive variants in exosome components *EXOSC3*, *EXOSC8*, and *RBM7* cause various constellations of pontocerebellar hypoplasia (PCH), spinal muscular atrophy (SMA), and central nervous system demyelination. Here, we report on four unrelated affected individuals with recessive variants in *EXOSC9* and the effect of the variants on the function of the RNA exosome *in vitro* in affected individuals' fibroblasts and skeletal muscle and *in vivo* in zebrafish. The clinical presentation was severe, early-onset, progressive SMA-like motor neuronopathy, cerebellar atrophy, and in one affected individual, congenital fractures of the long bones. Three affected individuals of different ethnicity carried the homozygous c.41T>C (p.Leu14Pro) variant, whereas one affected individual was compound heterozygous for c.41T>C (p.Leu14Pro) and c.481C>T (p.Arg161*). We detected reduced EXOSC9 in fibroblasts and skeletal muscle and observed a reduction of the whole multi-subunit exosome complex on blue-native polyacrylamide gel electrophoresis. RNA sequencing of fibroblasts and skeletal muscle detected significant >2-fold changes in genes involved in neuronal development and cerebellar and motor neuron degeneration, demonstrating the widespread effect of the variants. Morpholino oligonucleotide knockdown and CRISPR/Cas9-mediated mutagenesis of *exosc9* in zebrafish recapitulated aspects of the human phenotype, as they have in other zebrafish models of exosomal disease. Specifically, portions of the cerebellum and hindbrain were absent, and motor neurons failed to develop and migrate properly. In summary, we show that variants in *EXOSC9* result in a neurological syndrome combining cerebellar atrophy and spinal motoneuronopathy, thus expanding the list of human exosomopathies.

Introduction

The RNA exosome is a multi-protein complex that plays a vital role in gene expression via processing and degradation of mRNA.[1,2] The exosome is composed of nine subunits (EXOSC1–EXOSC9) forming a two-layered ring and is conserved in all eukaryotes.[3] EXOSC4–EXOSC9 form the core, a hexamer channel through which the RNA passes, and EXOSC1–EXOSC3 make up the cap of the exosomal ring for RNA recognition and binding.[4,5] The exosome in the nucleus processes precursor RNA and degrades precursor species, cryptic transcripts, and unspliced RNAs,[6–11] whereas in the cytoplasm, the exosome degrades defective transcripts that have evaded nuclear degradation and AU-rich element-containing mRNAs (AREs).[12] Exosomal RNA degradation proceeds in the 3′-to-5′ direction and is associated with other proteins, such as EXOSC10, for catalytic activity and the nuclear exosome targeting (NEXT) complex, which binds and delivers some specific non-coding RNAs to the exosome for degradation.[13–15]

Recessive variants in *EXOSC3* (MIM: 606489) and *EXOSC8* (MIM: 606019), encoding EXOSC3 and EXOSC8, respectively, of the human exosome, cause pontocerebellar hypoplasia type 1 (PCH1) of variable severity with spinal muscular atrophy (SMA) and hypomyelination of the central nervous system (CNS).[16,17] *EXOSC3* variants account for about 40% of PCH1 cases worldwide,[18] suggesting further genetic heterogeneity. On the other hand, *EXOSC8* variants seem to be much less frequent, given that despite screening of *EXOSC8* variants in large PCH1 cohorts, only one report on two *EXOSC8* variants has

[1]Wellcome Trust Centre for Mitochondrial Research, Institute of Genetic Medicine, Newcastle University, Newcastle upon Tyne NE1 3BZ, UK; [2]Neuromuscular and Neurogenetic Disorders of Childhood Section, National Institute of Neurological Disorders and Stroke, National Institutes of Health, Bethesda, MD 20892, USA; [3]Department of Neuropediatrics and NeuroCure Clinical Research Center, Charité-Universitätsmedizin, Charitéplatz 1, 10117 Berlin, Germany; [4]Division of Neurology, Children's National Medical Center, Washington, DC 20010, USA; [5]Section of Medical Genetics, Department of Pediatric Subspecialties, Children's Specialized Hospital, King Fahad Medical City, Riyadh, Saudi Arabia; [6]Division of Pediatric Neurology, Department of Pediatrics, University of Florida College of Medicine, Gainesville, FL 32610, USA; [7]Department of Genetics, King Faisal Specialist Hospital and Research Center, Riyadh, Saudi Arabia; [8]GeneDx, Gaithersburg, MD 20877, USA; [9]Departments of Pediatrics and Medicine, Dalhousie University, Halifax, NS B3H 4R2, Canada; [10]Maritime Medical Genetics Service, IWK Health Centre, Halifax, NS B3K 6R8, Canada; [11]Division of Neurology, Nemours/DuPont Hospital for Children, Wilmington, DE 19803, USA; [12]Division of Genetics, Nemours/DuPont Hospital for Children, Wilmington, DE 19803, USA; [13]Department of Radiology, University of Florida College of Medicine, Gainesville, FL 32610, USA; [14]Center for Mendelian Genomics, Program in Medical and Population Genetics, Broad Institute of MIT and Harvard, Cambridge, MA 01242, USA
[15]These authors contributed equally to this work
[16]These authors contributed equally to this work
*Correspondence: rita.horvath@ncl.ac.uk
https://doi.org/10.1016/j.ajhg.2018.03.011.
© 2018 The Author(s). This is an open access article under the CC BY license (http://creativecommons.org/licenses/by/4.0/).

been published to date,[17] leaving over 50% of PCH1 cases still unsolved. We have recently reported an individual who is affected by SMA without complex CNS involvement and who carries a homozygous variant in *RBM7* (MIM: 612413), which is a component of the NEXT complex and has been shown to interact with the exosome directly.[19] The *RBM7* variant led to a reduction in the steady-state levels of the exosome complex proteins and subsequently caused abnormal mRNA metabolism, resulting in aberrant gene expression and splicing or degradation of several coding and non-coding RNA species, which might explain the complex neuronal abnormalities similar to the primary exosomal conditions. Morpholino knockdown of either *exosc3*, *exosc8*, or *rbm7* in zebrafish results in developmental delay with defects in the motor neurons and cerebellum,[19] affecting neuronal systems similar to those in affected individuals. Thus, the zebrafish is a useful model for the elucidation of rare exosomal protein diseases.

Here, we present four unrelated individuals who are affected by a disorder closely related to PCH1 and who carry autosomal-recessive causative variants in *EXOSC9*. We report further insights into the pathomechanism of exosomal disease in human cells as well as in CRISPR/Cas9 and morpholino oligonucleotide zebrafish models.

Material and Methods

Recruitment and Sample Collection

Four unrelated affected individuals were included in this study. Written informed consent for study procedures and photographs was obtained by a qualified investigator (protocol 12-N-0095 approved by the National Institute of Neurological Disorders and Stroke, National Institutes of Health; Charité institutional-review-board approval EA2/107/14; King Faisal Specialist Hospital & Research Center research advisory council no. 2121053; protocol 201400469 approved by the University of Florida). Medical history was obtained and clinical evaluation and muscle biopsy were performed as part of the standard neurologic evaluation. DNA, muscle, and skin biopsy samples were obtained according to standard procedures. Affected individuals 3:II-1 and 4:II-1 were identified through GeneMatcher.[20]

Homozygosity Mapping, Exome Sequencing, and Haplotype Analysis

Chromosomal microarray analysis was performed in affected individual 1:II-1 with both copy-number and single-nucleotide polymorphism (SNP) probes on a whole-genome array (Affymetrix CytoScan HD platform). Exome sequencing was performed on genomic DNA extracted from blood (affected individual 1:II-1) and saliva (affected individual 1:II-1 parents). Trio exome sequencing was performed through the NIH Intramural Sequencing Center with the Nimblegen SeqCap EZ Exome+UTR Library and Illumina HiSeq 2500 sequencing instruments. Variants were analyzed with Varsifter and searched for in dbSNP, the National Heart, Lung, and Blood Institute Exome Variant Server, and the Exome Aggregation Consortium (ExAC) Browser.[21,22] The *EXOSC9* variant was confirmed by Sanger sequencing in affected individual 1:II-1 and her parents.

Exonic sequences from affected individual 2:II-1 were enriched with the SureSelect V5 Human All Exon Kit (Agilent) and sequenced on a HiSeq 2000 machine (Illumina) as 101 bp paired-end fragments. FASTQ files were aligned to the human GRCh37.p11(UCSC Genome Browser hg19) reference sequence with the Burrows-Wheeler Aligner (BWA-MEM) v0.7.1.[23] Subsequently, variant VCF files were generated for all exons ± 20 bp flanking regions with the Genome Analysis Toolkit (GATK) v3.8 software package[24] and sent to the MutationTaster2 software for assessment of potential pathogenicity.[25] Variants were filtered for a recessive model and removed if they occurred in the homozygous state either in the ExAC Browser in >20 cases or in the 1000 Genomes Project in >10 cases. All relevant variants were inspected visually with the Integrative Genomics Viewer (IGV), and their segregation was verified by Sanger sequencing with gene-specific oligonucleotide primers and the BigDye (Applied Biosystems) protocol on an ABI3500 Genetic Analyzer (ThermoFisher Scientific). For verification of the *EXOSC9* c.41T>C (p.Leu14Pro) and the c.481C>T (p.Arg161*) variants (GenBank: NG_029848.1) we analyzed the PCR-products generated with the oligonucleotide primer pairs 5′-gcccaagccatttcccattt-3′ (forward) and 5′-tcagtccacaccttgagacc-3′ (reverse) and 5′-cctgataaatagccactggttgt-3′ (forward) and 5′-tcctggttcacataggagct-3′ (reverse).

Whole-exome sequencing (WES) was performed in affected individual 3:II-1 with an Agilent SureSelect All Exons V5 (50 Mb) capture kit (Agilent Technologies) for library preparation. An Illumina HiSeq 2500 (Illumina) was used for paired-end 100 bp sequencing. Sequence alignment, indexing of the reference genome (hg19), variant calling, and annotation used a pipeline based on BWA, SAMtools, GATK (see Web Resources), and ANNOVAR. Variants were annotated with a combination of public knowledge databases available from the ANNOVAR package and in-house databases, which included collections of previously published Saudi disease-causing variants. Variants were interpreted with a previously described in-house variant interpretation pipeline.[26] The *EXOSC9* variant was confirmed by Sanger sequencing in affected individual 3:II-1 and her parents.

Lastly, with genomic DNA from affected individual 4:II-1, the exonic regions and flanking splice junctions of the genome were captured with the IDT xGen Exome Research Panel v1.0. Massively parallel (NextGen) sequencing was done on an Illumina system with 100 bp or greater paired-end reads. Reads were aligned to human genome build hg19 (UCSC Genome Browser) and analyzed for sequence variants with a custom-developed analysis tool. Additional sequencing technology and a variant interpretation protocol have been previously described.[27] The general assertion criteria for variant classification are publicly available on the GeneDx ClinVar submission page.

Haplotype analysis was determined by the identification of shared regions of homozygous variants from each of the affected individuals with the homozygous *EXOSC9* variant at chr4: 122,722,620. Only variants that were called as high confidence by their respective pipelines were included for analysis, and regardless of pipeline, only non-indel SNVs with at least 10× coverage were considered. Specifically, the largest extent of the shared runs of homozygosity (ROH) haplotype was determined by extending the ROHs from the variant in each direction until the first high-confidence heterozygote. The region was then trimmed back from those outer boundaries to the first high-confidence shared homozygote. We then intersected the three resulting

regions to determine the shared haplotype. To further assess possible shared ancestry, we performed principal-component analysis (PCA) and k-nearest neighbor (knn) classification on exome SNPs from the four individuals against random representative samples from the 1000 Genomes Project phase 3 super-populations plus a GeneDx-sequenced Middle Eastern population as previously described by Lake et al.[28]

Cell Culture

Fibroblasts of affected individuals carrying the homozygous c.41T>C in *EXOSC9* (individual 1:II-1), the homozygous c.5C>T in *EXOSC8*,[17] the homozygous c.92G>C in *EXOSC3*,[29] homozygous c.236C > G in *RBM7*,[19] and controls were grown in high-glucose Dulbecco's modified Eagle's medium (Sigma) supplemented with 10% fetal bovine serum and 1% penicillin and streptomycin.

Immunoblotting

Aliquots of total protein (30 μg) were loaded on 4%–12% SDS-polyacrylamide gels (NuPAGE 4%–12% Bis-Tris Protein Gels, ThermoFisher Scientific), transferred to a PVDF membrane with an iBlot2 PVDF Mini transfer stack (ThermoFisher Scientific), and subsequently probed with a polyclonal antibody recognizing EXOSC8 (Proteintech, 1:500), EXOSC3 (Proteintech, 1:1,000), EXOSC9 (Abcam ab156686, 1:1,000), RBM7 (Abcam ab84116, 1:500), β-actin (Sigma A1978, 1:2,000), and α-tubulin (Invitrogen A11126, 1:2,000).

Blue Native Polyacrylamide Gel Electrophoresis (BN-PAGE)

The BN-PAGE procedure for detecting the exosome complex has been adapted from Fasken et al.[30] Fibroblast pellets were re-suspended in cold BN-PAGE lysis buffer (10 mM Tris-HCl [pH 8]; 150 mM NaCl; 0.1% NP40, supplemented with protease inhibitors) and lysed on ice for 30 min. Lysates were centrifuged at 17,000 × *g* for 10 min, and 50–100 μg total protein of the supernatant was loaded on pre-cast BN-PAGE gels. NativePAGE Novex 3–12% Bis-Tris Protein Gels, NativePAGE Running Buffer, Cathode Buffer Additive, 4× Sample Buffer, and 5% G-250 Sample Additive (all ThermoFisher Scientific) were utilized for electrophoresis. Transfer and antibody detection were performed as described above.

RNA Isolation, RT-PCR, and RNA Sequencing in Affected Individuals' Fibroblasts and Muscle Samples

A BGI SEQ-500 SE50 sequencing library was prepared from total muscle RNA and sequenced at a depth of 60 Mio paired-end fragments on a SEQ-500 machine.[31] Total RNA was isolated in triplicate from primary fibroblast cell lines using the mirVana miRNA Isolation Kit (Ambion) and DNAse treated with the DNA-free DNA Removal Kit (Ambion). RNA sequencing (RNA-seq) libraries were prepared with Illumina TruSeq Stranded polyA enriched RNA with Ribo-Zero Human kit and were sequenced on an Illumina HiSeq 2500 platform according to paired-end protocol. Control muscle RNA sequences were obtained as described previously.[32] The quality of sequencing reads was checked with FastQC. Reads were aligned with the STAR (v2.5.2b) aligner and the two-pass protocol that is outlined in GATK documentation. Number of reads mapped to Ensembl GRCh38 v86 genes was counted with HTSeq-count.[33] Differentially expressed genes between affected and control individuals were identified with Bionconductor package DESeq2.[34] Genes with a false-discovery rate ≤ 0.1 and a log2 fold change ≥ 1 were considered differentially expressed. Gene-set enrichment analysis for gene ontology terms was performed with the ConsensusPathDB (CPDB) web tool.

Zebrafish Strains and Husbandry

All zebrafish used in this study were the *golden* strain, except where transgenic Islet1:GFP zebrafish were used for imaging motor neurons as described by Westerfield et al.[35] All procedures carried out on zebrafish were regulated by the UK Home Office.

sgRNA Synthesis

Crisprscan[36] was used to identify a target site in exon 3 of *exosc9* in zebrafish. Single guide RNA (sgRNA) was produced as described elsewhere.[37] An oligonucleotide with a T7 promoter, *exosc9* target sequence, and a complementary sequence (3′-taatacgactcacta taGGGGGGCGTGAATCTTTTGGgttttagagctagaa-5′) was annealed to a bottom strand "ultramer" oligo (3′-AAAAGCACCGACTCG GTGCCACTTTTTCAAGTTGATAACGG ACTAGCCTTATTTTAACT TGCTATTTCTAGCTCTAAAAC-5′) in a thermocycler, and extension of the oligonucleotides was catalyzed by DNA polymerase (MyTaq) to form the template oligonucleotide for sgRNA synthesis.[38] The sgRNA template oligonucleotide was purified with a Qiagen PCR Purification Kit. sgRNA was synthesized from the sgRNA oligonucleotide template with the MEGAshortscript T7 Kit (Ambion), purified with the mirVana RNA Isolation Kit (ThermoFisher Scientific), and stored at −80°C until required for injection.

Injection of Morpholino and sgRNA and Cas9

A splice-blocking morpholino for the boundary between intron 1 and exon 2 of zebrafish *exosc9* was used (Genetools, 3′-actttatctgtg taccgttttgtagCGCTTAGATGGGAGACAGACATACG-5′). Before injection, the morpholino and sgRNA was prepared. The sgRNA was diluted to 300 ng/μL with 2 μM Cas9 protein (NEB), 2M KCl, and 0.05% phenol red and heated to 37°C for 5 min. The morpholino was diluted in Danieau solution (0.4 mM $MgSO_4$, 58 mM NaCl, 0.7 mM KCl, 5 mM HEPES, 0.6 mM $Ca[NO_3]_2$ [pH 7.6]) with phenol red and was heated to 65°C for 5 min.[39] Freshly laid embryos were injected up until the two-cell stage with 1 nL of morpholino (20 ng) or 1 nL of guide RNA (gRNA). At least three clutches of embryos from different parents were used for each experiment.

RNA Isolation and RT-PCR in Zebrafish Embryos

Trizol (ThermoFisher Scientific) was used to isolate RNA from zebrafish embryos according to the manufacturer's instructions. Approximately 30 embryos for each experimental group were pooled for RNA isolation. The SuperScript III First-Strand Synthesis System (ThermoFisher Scientific) was used to produce cDNA from the isolated RNA according to the manufacturer's instructions. RT-PCR was performed with the primers listed in Table S1.

Genomic DNA Extraction and PCR in Zebrafish Embryos

Genomic DNA was extracted from single embryos with the "hotSHOT" technique.[40] Embryos were lysed in 20 μL of 50 mM NaOH (Sigma-Aldrich) for 30 min at 95°C during periodic vortexing. The lysate was then neutralized with 20 μL 100 mM Tris-HCl (Sigma-Aldrich). PCR was then performed on the lysates with the primers listed in Table S1.

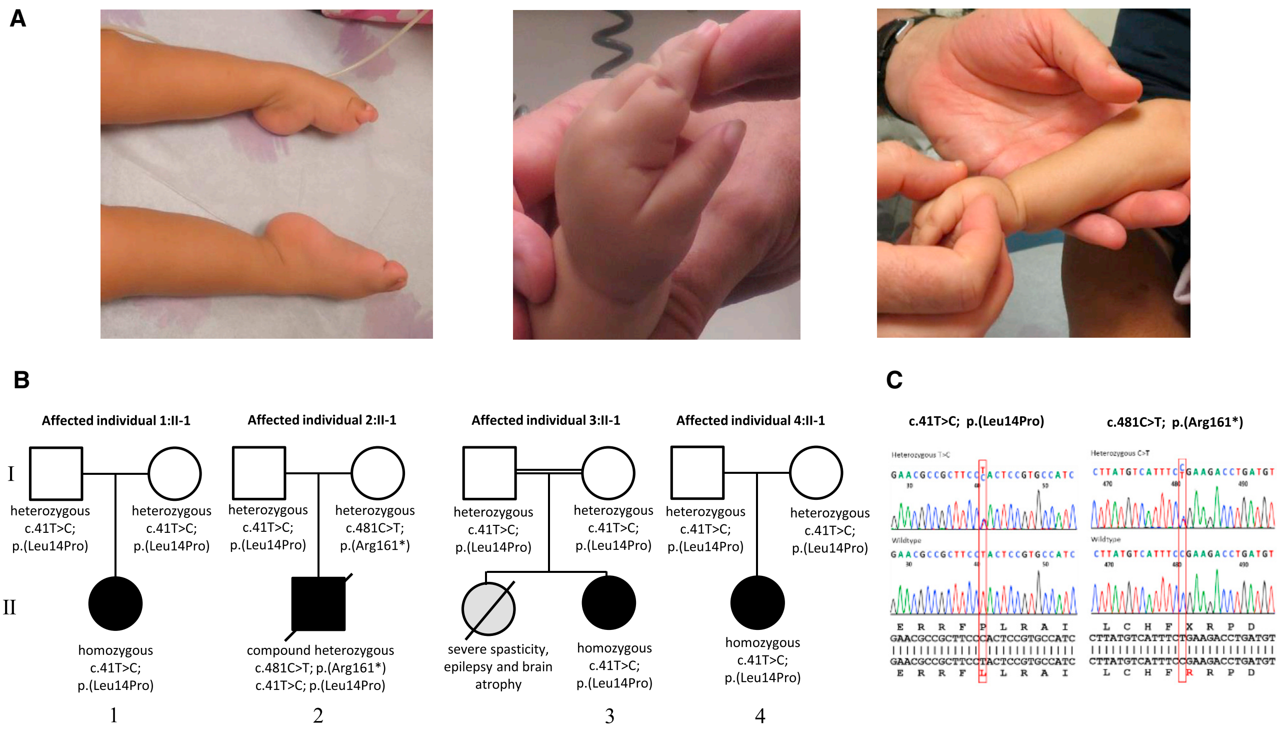

Figure 1. Clinical Presentation and Pedigrees
(A) Clinical photos of affected individual 1:II-1. She had multiple joint contractures. Hands were fisted with thumbs adducted, and feet were maintained in an extended cavo-varus position.
(B) Segregation of the *EXOSC9* sequence variants in the four pedigrees.
(C) Electropherograms of the *EXOSC9* sequence variants identified in this study.

Cloning and Sequencing of Crispant Zebrafish Embryos

Crispant embryos would be expected to be mosaic. To confirm that the mutation had occurred and to get a rate of mutagenesis, we cloned the heterogeneous PCR products from individual embryos into a vector so that individual mutations could be sequenced. The PCR products were ligated into the pGEM-T easy vector (Promega) according to the manufacturer's instructions. JM109 High Efficiency Competent Cells (Promega) were transformed with the ligated plasmid and plated on ampicillin resistant agar plates. Plates were incubated at 37°C overnight. Colony PCR was then performed on plasmids with standard pUC/M13 primers (Eurofins, Table S1). PCR products were sequenced subsequently.

Whole-Mount Immunofluorescence in Zebrafish Embryos

Zebrafish embryos were fixed overnight at 4°C in 4% paraformaldehyde in phosphate-buffered saline (PBS). If embryos were still in their chorion, they were dechorionated with pronase (Sigma-Aldrich) before fixation. Embryos were washed in PBS plus 0.1% Tween 20 (PBT, Sigma-Aldrich) and then permeabilized with acetone for 7 min at −20°C. If embryos were older than 48 hr post-fertilization (hpf), they were also permeabilized with collagenase (Roche) for 90 min. Embryos were then blocked in blocking solution (5% horse serum in PBT) for 1 hr. Embryos were incubated in the primary antibodies diluted in the blocking solution (SV-2, 1:200, Developmental Studies Hybridoma Bank; HuC, 5 μg/mL, ThermoFisher) overnight at 4°C. After being washed with PBT, embryos were incubated with secondary antibodies (anti-mouse Alexa Fluor 488 or anti-rabbit Alexa Fluor 594, Invitrogen) and diluted in blocking solution for 1 hr. Phalloidin and α-bungarotoxin (ThermoFisher Scientific) were conjugated to Alexa Fluor 594 and did not require any secondary antibodies. Immunofluorescent images were captured with a Nikon A1R confocal microscope with NIS-Elements software.

Results

Clinical Presentation

Individual 1:II-1 is a 28-month-old female of El Salvadorian descent (Figure 1; Video S1). Pregnancy was notable for reduced fetal movements. She was born at term by spontaneous vaginal delivery. Birth weight was 2.98 kg (10th–25th percentile), and length was 48 cm (25th percentile). There were no concerns regarding feeding or breathing in the neonatal period, and movements of the extremities were reported as normal. As an infant, she was noted to have poor head control and a soft cry. She was found to have congenital esotropia, which was managed with botulinum toxin A injection. By 8 months of age, she had increasing difficulty with lifting her arms and legs against gravity. Her strength declined further, which was exacerbated by periods of illness. By 9 months of age, she had lost the ability to vocalize. She rolled from back to side briefly but subsequently lost this skill. She had normal neonatal growth; however, by 21 months of age, her

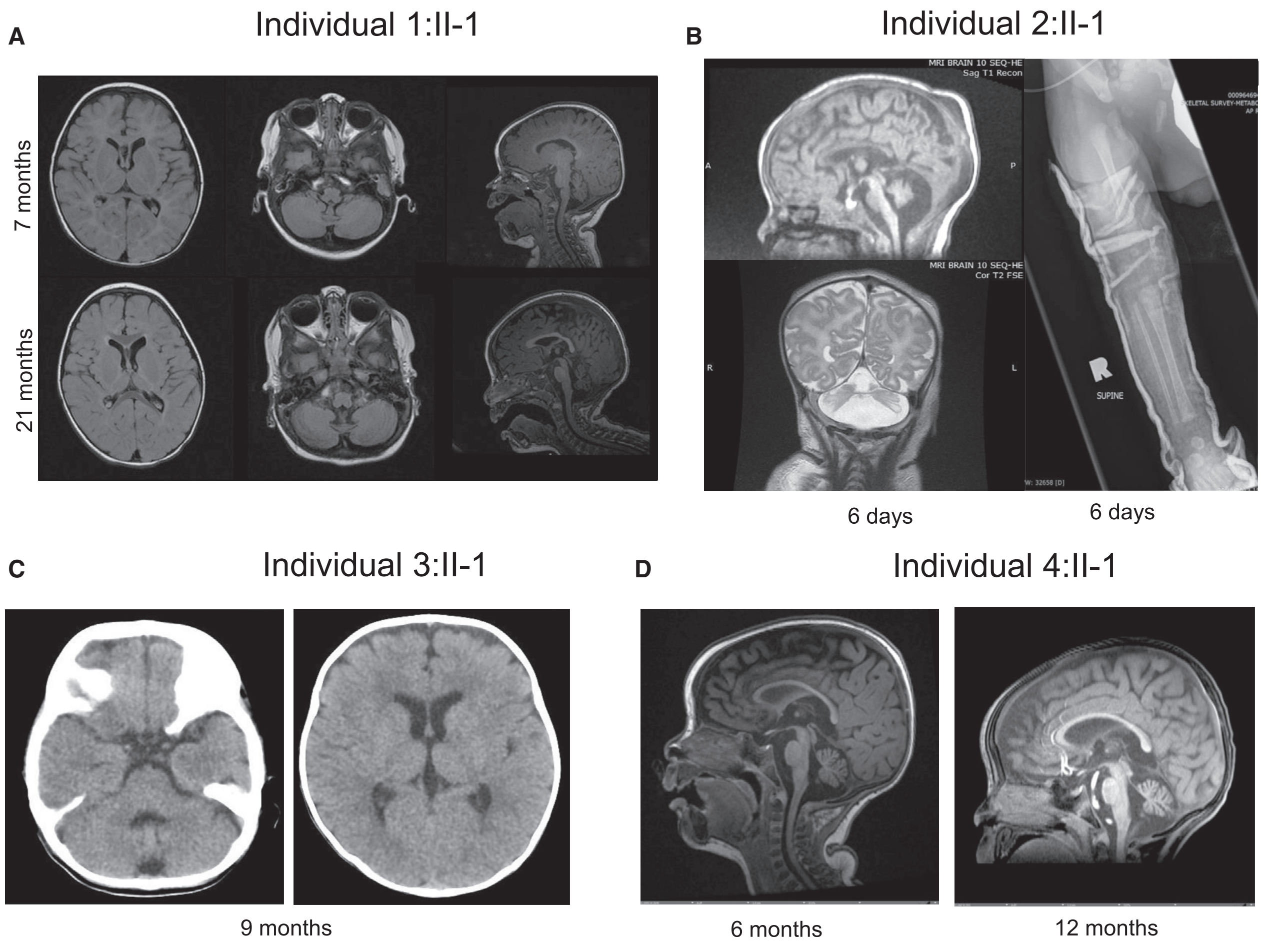

Figure 2. Radiographic Studies
(A) Axial and sagittal T1-weighted images of the brain from affected individual 1:II-1 at 7 months (top) and 21 months (bottom) of age show moderate cerebellar predominant volume loss, which appears mildly progressive between the two exams, as well as mild cerebral atrophy with resultant enlargement of the ventricles. There is no brainstem atrophy.
(B) Sagittal T1- and coronal T2-weighted images of the brain from affected individual 2:II-1 at 6 days of age show severe cerebellar and moderate cerebral and brainstem atrophy. A radiograph of the right leg also shows a mid-femoral fracture.
(C) Two axial CT images of the brain from affected individual 3:II-1 at 9 months of age show mild prominence of the sulci and ventricles reflecting mild atrophy both above and below the tentorium.
(D) Sagittal T1-weighted images of the brain from affected individual 4:II-1 at 6 months and 12 months of age show slightly progressive moderate cerebellar and cerebral atrophy. The brainstem is relatively spared.

weight, height, and head circumference were all below the third percentile. She had a history of recurrent pulmonary infections requiring hospitalizations, and nighttime non-invasive ventilation in the form of bilevel positive airway pressure (BiPAP) was initiated at 20 months of age. Examination at the age of 28 months revealed a high-arched palate and tongue fasciculations. Extraocular movements were full but were notable for broken pursuits and gaze-evoked nystagmus in horizontal and vertical directions. Although there was a tendency for downward deviation of the eyes, upward gaze was also observed. Occasional oromotor dyskinesia was noted on examination. She had severe axial and appendicular hypotonia and profound weakness, causing her to require full head support. She had no antigravity movements proximally in the upper and lower extremities. Wrist extension was antigravity at 3/5 (Medical Research Council-grade), whereas elbow flexion, knee extension, and finger extension were 2/5. Facial strength was relatively spared. Sensation appeared to be normal. Reflexes were absent throughout. Plantar response was flexor bilaterally. There were multiple joint contractures (Figure 1A). Hands were fisted with thumbs adducted, and feet were maintained in a cavo-varus position (Video S1).

Electromyography (EMG) and nerve conduction responses were consistent with an axonal motor neuronopathy. Muscle ultrasound revealed a pronounced streak-like and mixed increase in echogenicity with widespread evidence of active fasciculations, consistent with advanced neurogenic changes in the muscle in a non-length-dependent manner. Brain MRI performed at the age of 7 months revealed mild cerebellar atrophy with a normal-appearing pons and no significant abnormalities of the cerebral white matter or the basal ganglia (Figure 2A). A repeat brain MRI at 21 months of age

revealed a mild progression of the cerebellar atrophy (Figure 2A). Muscle biopsy performed at 15 months of age demonstrated abundant, very small fibers, often in groups and intermixed with hypertrophic fibers, consistent with denervation and thus suggestive of a neurogenic process. Lysosomal enzymes, plasma amino acids, carbohydrate transferrin, coenzyme Q10, and plasma lactate levels were normal. No seizures have been observed, and a routine electroencephalogram (EEG) performed at 5 months of age did not reveal any epileptiform activity. Family history was non-contributory, and the family denied known consanguinity. Extensive genetic testing for congenital muscle diseases, neuronal ceroid-lipofuscinoses, and *SMN1* were negative.

Individual 2:II-1 was the first child of non-consanguineous, healthy parents of African-Canadian and Jamaican descent (Figure 1B). During pregnancy, reduced fetal movements, growth retardation, and oligohydramnios were noted. He was born at term with several congenital fractures, including fractures of the bilateral femurs and one fracture of the humerus (Figure 2B). Examination findings were consistent with arthrogryposis multiplex congenita with hip and hand involvement, severe hypotonia, and respiratory insufficiency. Dysmorphic features, including hypertelorism, prominent epicanthic folds, low-set ears, prominent lips, and a short neck, were noted. Sclerae were described as blueish, and skin appeared redundant. As a result of swallowing difficulties, he required gastric-tube feeding. Brain MRI performed at 1 week of age showed cerebellar atrophy, generalized cerebral atrophy, and possibly delayed myelination (Figure 2B). Muscle biopsy findings, including fiber-type grouping, grouped fiber atrophy, and type 1 fiber hypertrophy, were suggestive of a motor neuronopathy. EMG was not performed. Genetic testing for deletions in *SMN1* and an SMA-related gene panel were negative. Chromosomal microarray analysis, methylation analysis for Prader-Willi syndrome, myotonic dystrophy testing, and metabolic screening were also negative. Individual 2 died of respiratory failure at the age of 15 months.

Individual 3:II-1 is a 4.5-year-old female born to consanguineous parents of Saudi-Arabian descent. She was noted at birth to have hypotonia, a poor cry, and difficulties with feeding. Subsequently, she was diagnosed with microcephaly and failure to thrive, and she was noted to have severe developmental delay. At the age of 5 months she was noted to have seizures, which were partially controlled by anti-epileptic drugs. Clinical examination at the age of 18 months showed minor dysmorphic facial features. She was unable to visually track objects. She was not vocalizing and appeared to have receptive language difficulties. There was axial hypotonia, generalized weakness of the upper and lower limbs, and an apparent increased tone of the extremities with evidence of exaggerated deep tendon reflexes. She underwent brain imaging (computed tomography [CT]), which revealed minimal cortical atrophy (Figure 2C). Family history is significant for an older sister who died at the age of 8 years and who reportedly manifested severe spasticity and epilepsy. DNA from the sister was unavailable for diagnostic testing. Routine biochemistry, renal profile, total creatine phosphokinase, ammonia, plasma amino acids, very-long-chain fatty acids, and urine for organic acids were normal. Chromosomal microarray was normal. EMG and muscle biopsy were not performed.

Individual 4:II-1 is a 19-month-old female born to non-consanguineous parents of African, European and Filipino ancestry. Pregnancy was uncomplicated, and delivery was induced at 40+ weeks. She was born via Caesarean section with vacuum assistance because of fetal distress. Birth weight was 2.81 kg. She was found to have mild jaundice and congenital nystagmus. At 2 weeks of age she was noted to have poor head control and a weak cry. She had excessive oral secretions and difficulty with clearing her airway. Over time, her head control slowly improved. She started to visually track and follow at the age of 6–7 months. Her weight gain had stagnated between 6 and 12 months, but by 19 months her weight was above the 50th percentile. Her length had been between the 25th and 75th percentiles and her head circumference between the 10th and 25th percentiles. On examination at the age of 12 months she was found to have a moderately high-arched palate and a weak cry. Extraocular movements were full, but she had a recurrent intermittent horizontal nystagmus. She had diffuse hypotonia and weakness with poor truncal control, requiring head support. She was unable to sit and reach for objects. Her muscles were atrophic, and she had minimal antigravity movements in all extremities. Sensation appeared to be normal. Reflexes were 2+ in upper extremities, 1+ at the patellae, and absent at ankles. Plantar response was flexor.

EMG was suggestive of a motor neuropathy or motor neuronopathy. Brain MRI at the age of 6 months showed cerebellar atrophy but a normal-appearing pons. Repeat brain MRI at 12 months revealed a slight progression of the cerebellar atrophy (Figure 2D). Muscle biopsy was suggestive of neurogenic atrophy with fiber-type grouping. Sural nerve biopsy was normal. EEG studies performed on multiple occasions were mostly read as normal, though one earlier study was interpreted as showing myoclonic discharges. Creatine kinase was borderline at 147 U/L (reference range < 143 U/L). Cerebrospinal fluid studies for neurotransmitters were normal. Extensive genetic testing, including a chromosomal microarray, Prader-Willi methylation testing, *SMN1* deletion testing, *IGHMBP2* sequencing, and a neuromuscular disorder gene panel, was negative.

The clinical presentation seen in these four unrelated affected individuals of early-onset, rapidly progressive weakness and respiratory impairment combined with the presence of cerebellar atrophy and a motor neuronopathy suggests a condition along the PCH1-related disease spectrum.

Identification of *EXOSC9* Variants

SNP array testing in individual 1:II-1 revealed one isolated long contiguous stretch of homozygosity of approximately 14.0 Mb on chromosome 4 (117,649,360–131,644,865). Exome sequencing identified a rare homozygous variant, c.41T>C (p.Leu14Pro), in *EXOSC9* (MIM: 606180; GenBank: NG_029848.1) within the region of homozygosity. This mutation is predicted to cause a disruption in the first alpha helix of EXOSC9.[41] The parents of individual 1:II-1 were found to be heterozygous for the variant, consistent with autosomal-recessive inheritance (Figure 1B). The variant is a rare SNP (dbSNP: rs139632595) and has been reported six times in heterozygous state in the ExAC Browser in individuals from African descent with an allele frequency of 4.947×10^{-5}. This variant was neither reported in individuals from Hispanic descent nor found in a homozygous state.

WES of individual 2:II-1 revealed the same c.41T>C variant as in individual 1:II-1 in compound heterozygosity with a *EXOSC9* c.481C>T variant that leads to a premature stop of the protein (p.Arg161*). The c.481C>T variant was listed three times in the ExAC Browser in the heterozygous state. To exclude variants in genes that are known to be associated with either SMA or with muscle diseases, we specifically screened the variant VCF files for variants therein but found none. Sanger sequencing confirmed compound heterozygosity (Figure 1).

Individuals 3:II-1 and 4:II-1 were subsequently identified to carry the same *EXOSC9* c.41T>C in homozygosity.

Haplotype Analysis

The shared haplotype analysis revealed a common haplotype of 800 kb at approximately chr4: 122,400,000–123,200,000, encompassing *ANXA5*, *TMEM155*, *EXOSC9*, *BBS7*, *TRPC3*, and *KIAA1109*. The largest homozygous block was identified in individual 1:II-1 and extended approximately 11 Mb, whereas the other two homozygotes, individuals 3:II-1 and 4:II-1, showed ROHs of only approximately 1 Mb. Our common haplotype was estimated at 800 kb. Using the approximation given by Ying et al.,[42] we can estimate a common ancestor approximately 125 generations ago. The results of ancestry PCA are consistent with self-reported ethnicities. Although these self-reported ethnicities are superficially divergent, PCA shows that these individuals do potentially share some common ancestry, most likely northern or eastern African. Additionally, compared with individuals in the primary super-population clusters, all four individuals are relative outliers in the PCA space (Figure S1).

The Exosome Complex Is Reduced in Skeletal Muscle and Fibroblasts

Immunoblotting for components of the exosome complex was performed on cultured fibroblasts of individual 1:II-1 and skeletal muscle of individual 2:II-1. EXOSC9 was less abundant in both affected fibroblasts and skeletal muscle than in controls. Additionally, in fibroblasts from individuals harboring mutations in *EXOSC3*, *EXOSC8*, and *RBM7*, EXOSC9 was reduced; the reduction was most pronounced in cells from the *EXOSC3* and *EXOSC8* cell lines (Figure 3A). These data suggest that a primary reduction in one component of the exosome complex, or in a related protein such as RBM7, leads to destabilization of the whole complex. BN-PAGE with protein lysates from affected individual's fibroblasts probed with an antibody for EXOSC3 supported this hypothesis. Variants in EXOSC3, EXOSC8, EXOSC9, and RBM7 resulted in reduction of the exosome complex irrespective of which subunit carried the primary variant (Figure 3B).

Variants in *EXOSC9* Result in Abnormal RNA Metabolism

To study the effect of *EXOSC9* variants on gene expression, we performed RNA-seq on RNA collected from cultured affected individuals' fibroblasts (individual 1:II-1) and skeletal muscle (individuals 1:II-1 and 2:II-1). In the fibroblasts of individual 1:II-1, 69 genes (22 containing AREs) showed a significant increase and 138 genes (35 containing AREs) showed significantly less RNA expression than control individuals (Figure 4A; Table S4). Many of the enriched Gene Ontology (GO) terms from both the significantly increased and decreased genes described cellular and embryonic developmental processes of the neuronal system (Figure 4B). *EXOSC9* mRNA and mRNAs encoding other subunits of the exosome complex did not show a significant difference in expression. Previously, we showed that expression of *HOTAIR*, *HOXC6*, *HOXC8*, and *HOXC9* was significantly elevated in fibroblasts from individuals with variants in *EXOSC8* and *RBM7*. However, in fibroblasts from individuals with variants in *EXOSC9* and *EXOSC3*, only *HOXC8* expression was higher than in control fibroblasts; interestingly, increased *HOTAIR* mRNA seems to be specific to the *EXOSC8* and *RBM7* mutant cells (Figure 4D).

RNA-seq revealed that a high number of genes were significantly differentially expressed between muscle biopsies of individual 2:II-1 and control fibroblasts and fibroblasts of individual 1:II-1: 2,778 genes (497 containing AREs) were significantly upregulated and 2,864 (448 containing AREs) were downregulated in both individuals (Figure 4C; Table S4). Expression of several genes linked to motor neuronopathy and familiar amyotrophic lateral sclerosis (*EPHA4*, *IGHMBP2*, *VAPB*, *BICD2*, and *DYNC1H1*) or distal arthrogryposis (*MYH8*, *ZC4H2*, *MUSK*, *RAPSN*) were significantly upregulated or downregulated. In individual 2:II-1, who presented with congenital fractures, we detected a higher number of significant changes in gene expression than in individual 1:II-1. This included seven genes (*LIFR*, *TMEM38B*, *PLS3*, *NANS*, *SLC26A2*, *ALX4*, and *PLS3*) associated with skeletal dysplasia or bone disease, and four of them were ARE-containing genes with increased expression. A comparison of RNA expression between the fibroblasts and muscle of individual 2:II-1 showed that only 17 genes were

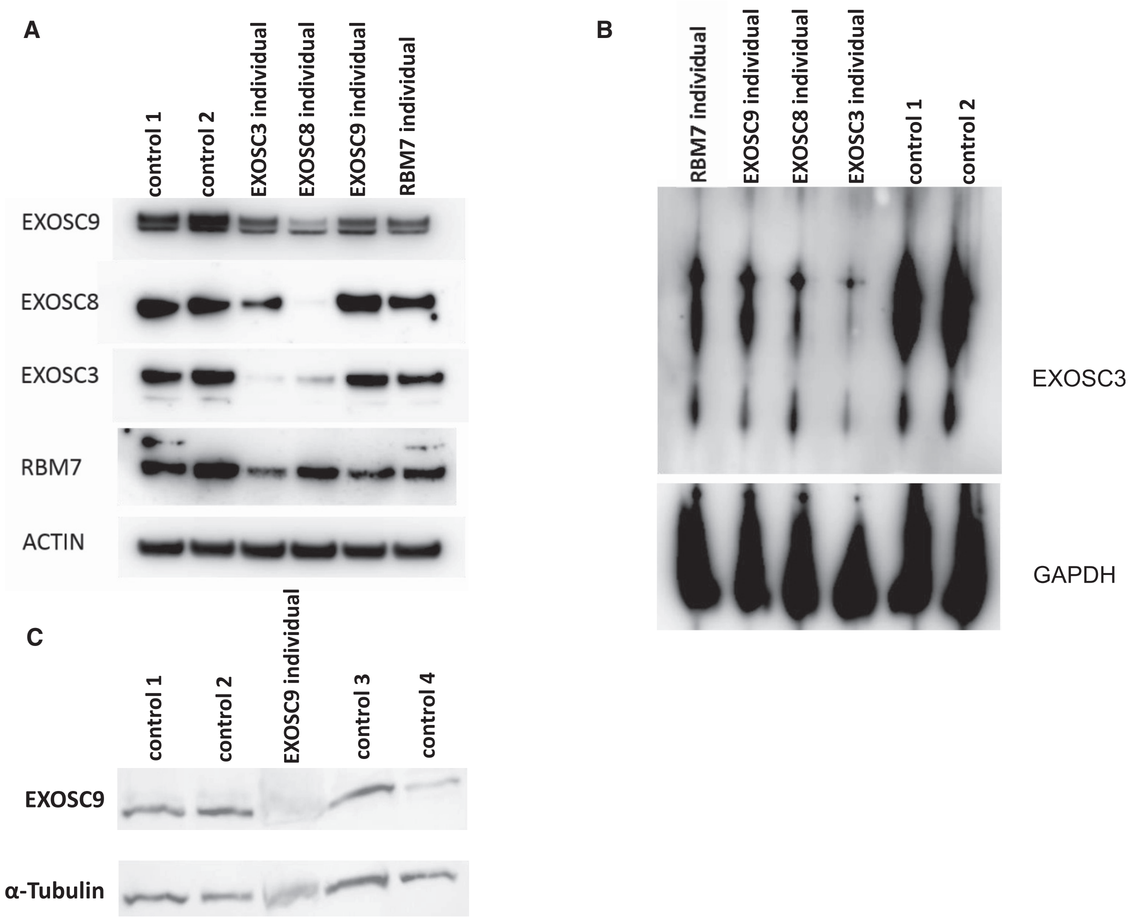

Figure 3. The Exosome Complex Is Reduced in Affected Individuals' Fibroblasts

(A) Immunoblotting of fibroblasts from affected individuals with variants in different components of the exosome complex (the homozygous c.92G>C in *EXOSC3*, the homozygous c.5C>T in *EXOSC8*, the homozygous c.41T>C in *EXOSC9* [individual 1:II-1], and the homozygous c.236C>G in *RBM7*) shows reduced EXOSC9, but other components of the exosome complex were also reduced. Actin was used as a loading control.

(B) Blue native polyacrylamide gel electrophoresis (BN-PAGE) shows that there is a reduction of the assembly of the whole exosome complex in affected individuals' fibroblasts. GAPDH was used as a loading control.

(C) Immunoblot on muscle extracts from affected individual 2:II-1 and four controls confirms that EXOSC9 was severely reduced in affected individual 2:II-1.

significantly upregulated and that 16 genes were significantly downregulated in both samples, suggesting tissue-specific differences.

Knockdown or Variants in *exosc9* Cause Developmental Defects in Zebrafish

Zebrafish have previously been used as model systems for investigating variants in exosome complex subunits[16,17] and associated proteins[19] and are consistently used for modeling the cerebellar, hindbrain, and motor neuron dysfunction observed in human disease. We concluded that zebrafish would therefore make a suitable *in vivo* disease model for the effects of reduced *exosc9* function for investigating whether a phenotype consistent with the other exosomal models would result.

Injection of morpholino oligonucleotides and the CRISPR/Cas9 system were used to knock down or induce variants, respectively, in *exosc9* in zebrafish embryos (Figure 5A). The *exosc9* morpholino oligonucleotides led to aberrant splicing of the *exosc9* transcript, which was confirmed via RT-PCR (Figure 5B), where morphant zebrafish had a retained intron that was confirmed by sequencing. In addition to the appearance of mis-spliced transcripts, the amount of wild-type (WT) *exosc9* transcript was reduced in injected embryos. The embryos injected with Cas9 and gRNA for *exosc9* would be expected to be mosaic; genomic DNA of cells would be a mixture of WT and various mutated forms of *exosc9* in varying proportions. To confirm mutagenesis in the crispants, PCR with primers flanking the sgRNA target area was performed on genomic DNA. The PCR product was then cloned into the pGEM-T easy vector and colony PCR, and sequencing was performed on individual clones. Sequencing showed that there was a variation in the amount of mutagenesis occurring and that there was a phenotype-genotype correlation (Figure 5D).

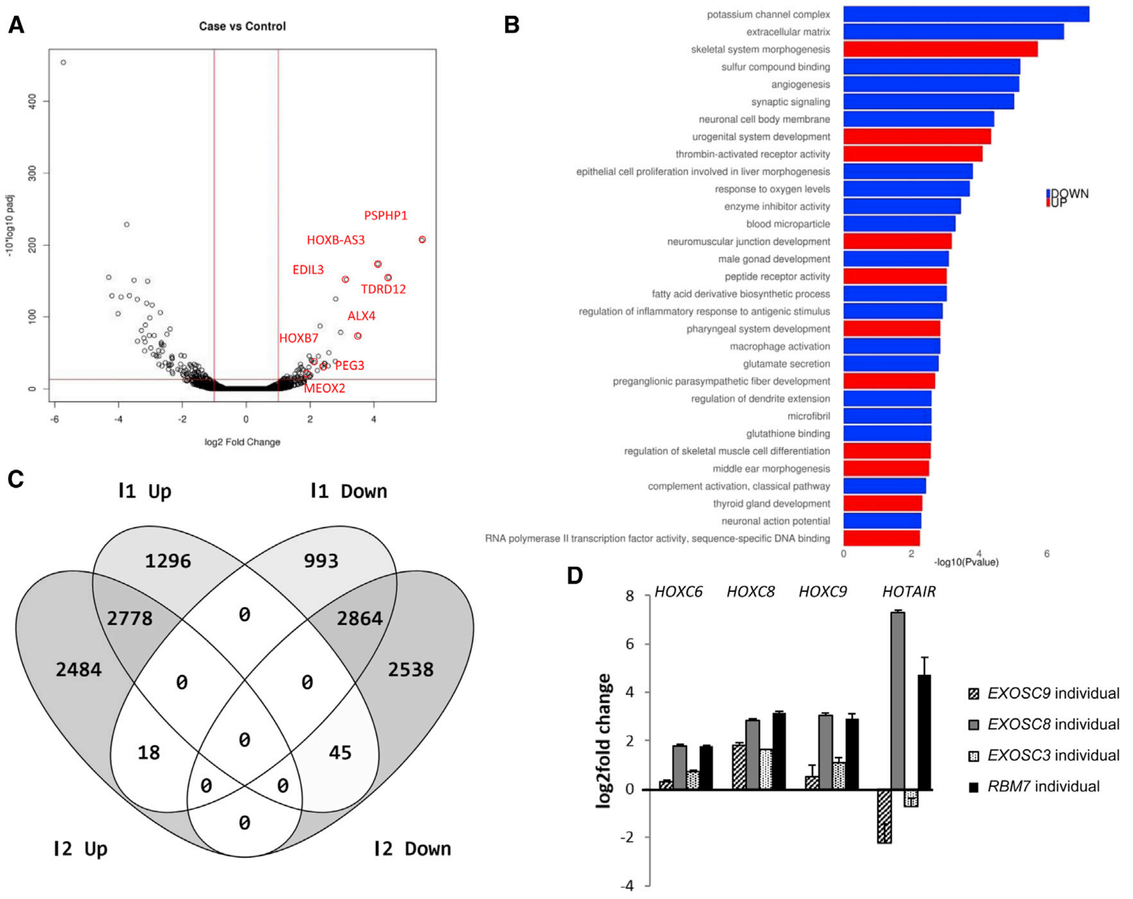

Figure 4. RNA-Seq and qPCR in Fibroblasts of Affected Individual 1:II-1 Carrying the Homozygous c.41T>C Sequence Variant in *EXOSC9*

(A) Differential expression analysis (DESeq2) detected 69 genes that were significantly upregulated and 138 that were significantly downregulated in the affected individuals' fibroblasts.

(B) Gene-set enrichment analysis of GO terms with differentially expressed genes in fibroblast RNA-seq.

(C) Comparison of the numbers of upregulated and downregulated genes in muscle biopsy specimens of RNA-seq between affected individual 1:II-1 and affected individual 2:II-1. 2,778 transcripts were upregulated in both affected individuals, whereas 2,864 transcripts were downregulated in both. 18 transcripts that were upregulated in affected individual 2:II-1 were downregulated in affected individual 1:II-1, and 45 transcripts were upregulated in affected individual 1:II-1 but downregulated in affected individual 2:II-1.

(D) Gene expression analysis of *HOXC6*, *HOXC8*, *HOXC9*, and *HOTAIR* through qRT-PCR in *EXOSC3*, *EXOSC8*, *EXOSC9*, and *RBM7* mutant fibroblasts. Results were normalized to the average values obtained from two control fibroblast lines.

Zebrafish injected with the morpholino oligonucleotide (morphants) and Cas9 and *exosc9* sgRNA (crispants) developed a similar range of morphological phenotypes (Figure 6A). Mildly affected embryos had smaller heads and eyes, whereas severely affected embryos had extremely small, sometimes absent, eyes, very small heads, and truncated bodies (Figure 6A). The relative distribution of phenotypes was also similar in morphants and crispants (Figure 6B).

In Zebrafish, *exosc9* Is Required for Brain and Neuromuscular Development

Next, we investigated whether *exosc9* was required for brain and neuromuscular development. Whole-mount immunofluorescence performed on 48 hpf *exosc9* morphants and crispants with an antibody against the neuronal marker, HuC, showed that the brain fails to properly develop (Figure 7A). In *exosc9* morphants and crispants, it was common for the brain to be misshapen and for the cerebellum and hindbrain to be absent. Morpholino knockdown and CRISPR/Cas9 mutagenesis of *exosc9* was also performed in the transgenic line of zebrafish, *Islet1:GFP*. This line of zebrafish produce green fluorescent protein in the motor neurons in the hindbrain.[43] In WT zebrafish, the cranial nerves are very distinct and can be visualized in the *Islet1:GFP* zebrafish. Cranial nerve V is split into two distinct parts, anterior (Va) and posterior (Vp).[43] In the *exosc9* morphants and crispants, it was common for only Va to be present. This again suggests that functional *exosc9* is required for the posterior part of the brain to develop in zebrafish (Figure 7B). Whole-mount immunofluorescence using an antibody against synaptic vesicle 2 (SV-2, presynaptic motor axons) and α-bungarotoxin (neuromuscular junctions) conjugated to Alexa Fluor 594

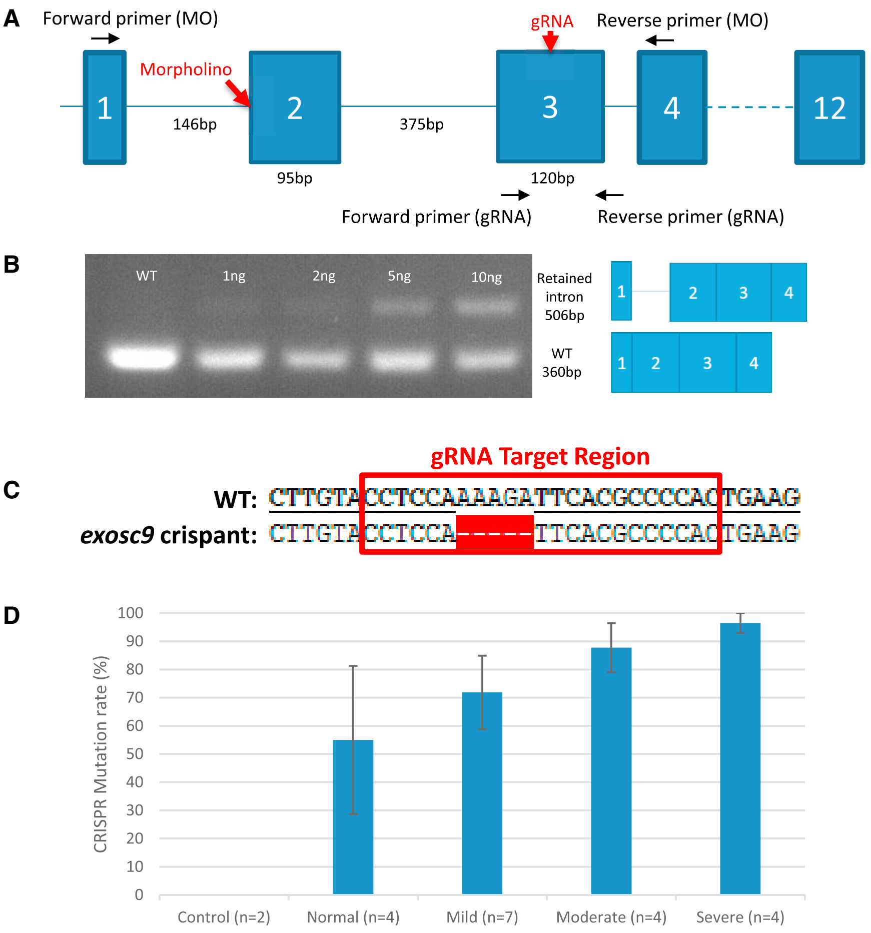

Figure 5. Strategies Targeting *exosc9* in Zebrafish

(A) Schematic of *exosc9* in zebrafish demonstrating the sites to where the morpholino, gRNA, and primers were targeted.

(B) RT-PCR of zebrafish morphants. The morpholino caused the retention of an intron and a reduction of the WT product in a dose-dependent fashion. The identity of the bands was confirmed by Sanger sequencing.

(C) The target sequence for *exosc9* gRNA and an example of a mutation found in a crispant.

(D) The mutation rate found in crispants of differing phenotypes.

showed that the neuromuscular junctions developed abnormally in the 48 hpf morphants and crispants (Figure 7C). In both morphants and crispants, the neuromuscular junctions appeared closer together and the motor axons failed to migrate properly to the neuromuscular junctions, indicating a primary pathfinding defect of the motor axons. Pathfinding defects in motor axons have been illustrated in other zebrafish models of neuronopathies—exosc3, exosc8, RBM7,[19] and SMA.[44] Phalloidin staining in 48 hpf morphants and crispants showed a reduced amount of muscle and damaged and misaligned myofibres (Figure 7D). Together, these results show that *exosc9* is also important in neuromuscular development.

Discussion

We report four independent affected individuals with an early-onset progressive axonal motor neuronopathy, resulting in severe weakness and respiratory impairment, in combination with cerebellar atrophy. All affected individuals harbor autosomal-recessive variants in *EXOSC9*, which encodes an exosomal protein. Individual 2:II-1 presented with congenital fractures and arthrogryposis at birth and subsequent symptom progression resulting in respiratory failure and death at 15 months of age. This individual carried the heterozygous null variant in combination with the missense variant, whereas the three other affected individuals carried this missense in homozygosity; this might explain the more severe clinical presentation in individual 2:II-1. Individual 1:II-1 and individual 4:II-1 showed milder phenotypes, starting with congenital esotropia and congenital nystagmus, respectively, and poor head control in a relatively normal neonatal course. Significant developmental delay, progressive muscle weakness, oculomotor dysfunction, and coordination difficulties became evident only after 6 months of age. In particular, the lower motor neuron symptoms subsequently progressed rapidly during the second year of life: these included limited spontaneous movement of the extremities, and individual 1:II-1 also required mechanical ventilation. Individual 3:II-1 had a more pronounced neonatal presentation with muscle hypotonia, weak cry, feeding difficulties, and early-onset seizures at the age of 5 months. The phenotype of individual 3:II-1 could be partially due to another, yet to be identified, recessive genetic disorder segregating in the family, given the parental consanguinity and the history of a sister who had severe spasticity and epilepsy and passed away at age 8 years. Unfortunately, DNA from the sister of individual 3:II-1 was not available for genetic testing.

The involvement of spinal motor neurons together with cerebellar atrophy, with or without pontine involvement, is a common feature in affected individuals with variants in different exosomal subunit (*EXOSC3*, *EXOSC8*, and *EXOSC9*) and belongs to the spectrum of PCH1-related disorders. Although these variants typically present with severe weakness precluding sitting in most cases, variants in *EXOSC3* in particular are now recognized to also include a milder presentation. However, CNS hypomyelination has thus far been noted only in affected individuals with

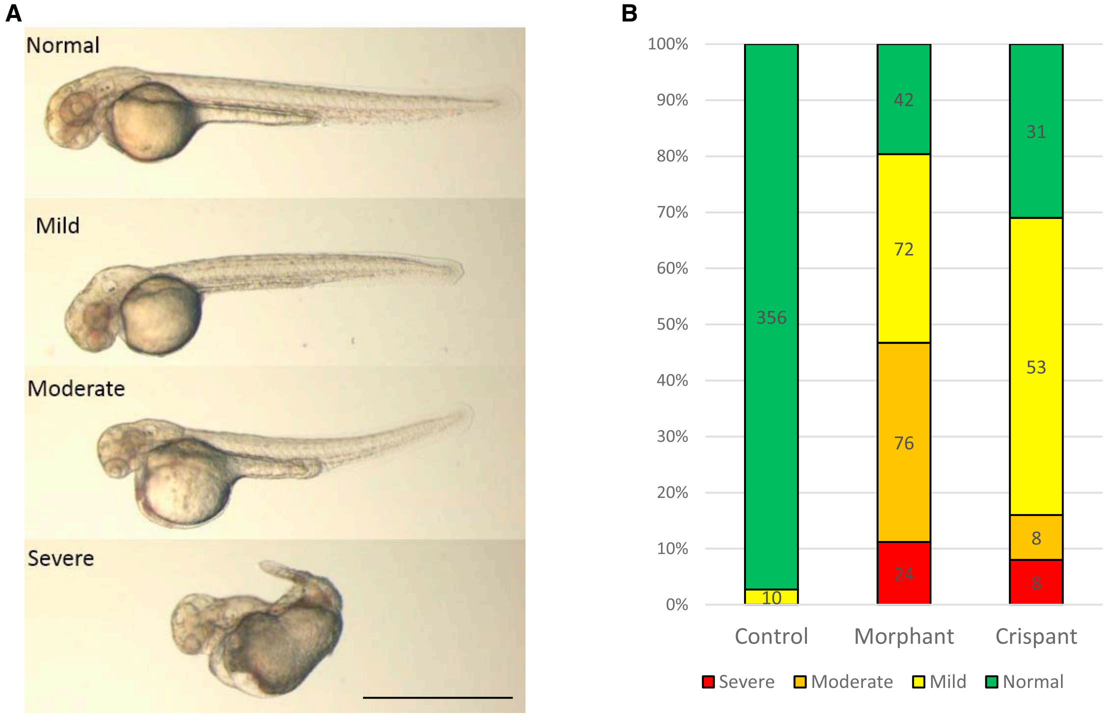

Figure 6. Knockdown of *exosc9* in Zebrafish Causes Abnormal Morphology
(A) Representative morphological scoring of morphant and crispant *exosc9* zebrafish embryos at 48 hpf. Normal, identical to uninjected control clutchmates; mild, smaller head and smaller eyes; severe, very small head, smaller or absent eyes, and misshapen body.
(B) Relative distribution of morphological phenotypes in *exosc9* morphants and crispants at 48 hpf. Scale bar represents 1 mm.

variants in *EXOSC8*. The presence of congenital symptoms in more severe exosomal protein defects suggests a developmental impairment; however, a progressive deterioration of motor skills in the first few months of life confirms that exosomal proteins are important not only for neuronal development but also for the survival of spinal motor and cerebellar neurons. Although the disease in our affected individuals fits into the PCH1 spectrum, the presence of congenital fractures in the severe case described here (individual 2:II-1) is reminiscent of congenital bone fractures with prenatal SMA caused by variants in subunits of the transcriptional coactivator complex, another disorder caused by abnormal RNA function.[45] Although not all affected individuals present with obvious pontocerebellar hypoplasia at birth, all affected individuals show cerebellar atrophy and signs of motor neuronopathy with onset in early childhood, suggesting the very characteristic involvement of the cerebellum and spinal motor neurons. Similar to PCH2, which results from variants in genes encoding subunits of the tRNA-splicing endonuclease complex (TSEN),[46] PCH1 has been also classified as PCH1A (*VRK1* variants), PCH1B (*EXOSC3* variants), and PCH1C (*EXOSC8* variants). We propose to classify the disease caused by *EXOSC9* variants as PCH1D. This would allow the characterization of the different phenotypes within each genotype.

In the four families reported here, we detected two different *EXOSC9* variants (c.41T>C and c.481C>T). Interestingly, haplotype analysis revealed a common haplotype shared among all three affected individuals homozygous for the c.41T>C variant. Given the milder phenotype of affected individuals carrying this homozygous variant, we suspect that its loss of function is not as severe as in the case of the nonsense variant c.481C>T. The presence of two severe nonsense variants in one individual would most likely result in intrauterine lethality, which has also been proposed for other exosomal protein variants in *EXOSC8*[17] and *EXOSC3*.[47] We noted that there has been no case of a complete loss of function in any of the exosomal components, thus reinforcing our hypothesis that such a defect would be cause intrauterine lethality.

We detected reduced levels of the protein EXOSC9 in two affected individuals, indicating a loss-of-function effect of the variants, as we have shown previously for *EXOSC8* and *RBM7* variants. Notably, the other subunits of the exosome (EXOSC3 and EXOSC8) were also reduced, suggesting that the exosome complex cannot be assembled or might be unstable if one of the subunits is primarily reduced. This notion was also supported by the detection of a lower total amount of the entire exosome complex by BN-PAGE in all cells from affected individuals with exosome variants. The individuals with *EXOSC8* and *EXOSC3* variants and severe phenotypes demonstrate the most pronounced reduction of the whole exosome and its subunits, whereas the reduction of the exosome was less pronounced in affected individuals with *EXOSC9* (individual 1:II-1) and *RBM7* variants. This pathophysiological concept would suggest that the disease manifestations

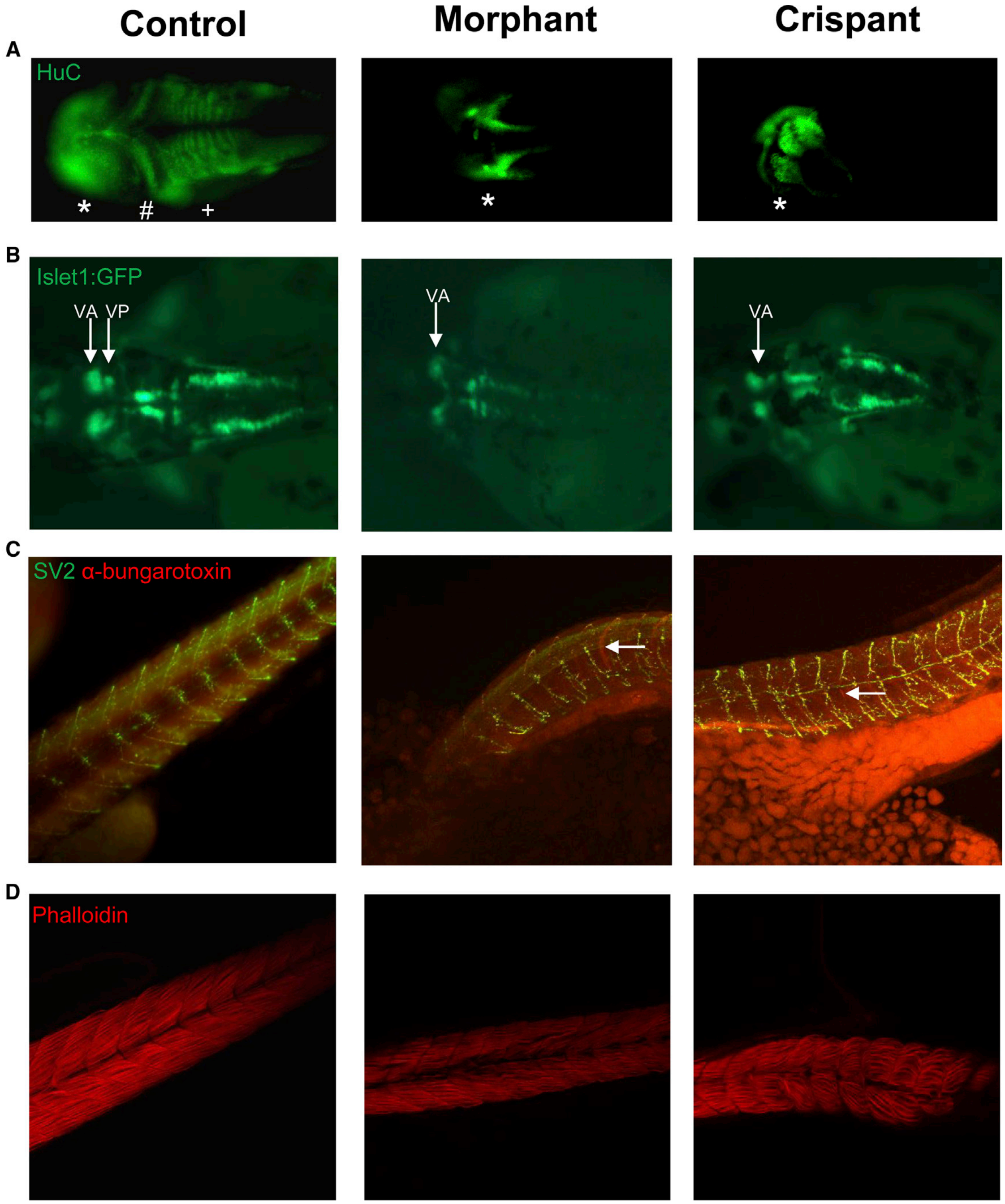

Figure 7. Knockdown of *exosc9* in Zebrafish Causes Abnormal Neuromuscular Development
(A) Whole-mount immunofluorescence of the pan-neuronal marker HuC shows that the midbrain (*) appears abnormal and the cerebellum (#) and hindbrain (+) are absent in representative *exosc9* morphants and crispants compared with uninjected controls.
(B) *Islet1:GFP* transgenic morphant and crispant zebrafish have absent cranial posterior nerve V (Vp).
(C) Whole-mount immunofluorescence of synaptic vesicle 2 (SV2, motorneurons, green) and α-bungarotoxin (neuromuscular junctions, red) shows that motoneurons and neuromuscular junctions failed to properly develop in *exosc9* morphants and crispants compared with uninjected controls (white arrows).
(D) Phalloidin staining shows that muscle failed to develop properly. Fibers were sparser, more spread out, and irregular in the *exosc9* morphants and crispants. All experiments were performed in 48 hpf zebrafish.

and their severity are more the result of the degree of overall exosome dysfunction rather than of a dysfunction of individual exosome components. This would be consistent with the similar nature of the "exosomopathies" as a group.

We performed RNA-seq analysis in fibroblasts and skeletal muscle of affected individuals with *EXOSC9* variants to investigate the effect of exosome impairment on the mRNA level. In fibroblasts, we detected significantly altered expression of genes involved in embryonic

development of neurons (*HOXB-A53*, *HOXB7*, and *HOXC8*) and genes encoding extracellular matrix proteins and potassium channels, but we did not detect other neuron-specific gene alterations, probably because we studied fibroblasts and not neurons. RNA-seq in muscle showed altered gene expression of a high number of genes, suggesting that all tissues might be affected by the exosome defect. Expression of *EXOSC9* and other genes encoding exosomal proteins was not different from control levels, suggesting that the downregulation of the exosome proteins is not due to reduced gene expression but is probably secondary to the instability of the holocomplex. Some of the genes contained AREs, suggesting that *EXOSC9* variants affect the degradation of AU-rich transcripts, which is one of the best-known functions of the exosome in the cytosol. However, changes in gene expression of many non-AU-rich genes suggest a more widespread exosome function, potentially involving splicing and other forms of gene expression regulation. Further studies are needed to explore the mechanism of altered RNA metabolism in neurons.

We studied reduced *exosc9* in an *in vivo* model by morpholino oligonucleotide knockdown and CRISPR/Cas9 mutagenesis in zebrafish embryos. Morpholino oligonucleotide knockdown is an established technique used for modeling diseases in zebrafish, although there has been recent criticism of the specificity of the morpholino oligonucleotides and suggestions of "off-target" effects.[48] To address this potential problem, we complementarily used CRISPR/Cas9 mutagenesis to produce mosaic "crispant" zebrafish[49] Knockdown and mutagenesis of *exosc9* produced similar phenotypes in zebrafish, which suggests that both methods specifically target and reduce *exosc9*, although we could not find any antibody that successfully identified any of the exosome components in zebrafish to prove that there was indeed a reduction at the protein level.

The phenotype caused by the reduction of *exosc9* in zebrafish is generally consistent with the phenotypic aspects (in particular, cerebellar defects and motor neuron pathology) seen in the four affected individuals. The phenotype of the *exosc9* morphants and crispants was also similar to that observed in *exosc3*, *exosc8*, and *rbm7* morphant zebrafish.[16,17,19] Interestingly, other zebrafish models of cerebellar hypoplasia and atrophy caused by abnormal RNA processing have a phenotype similar to that of the *exosc9* downregulated zebrafish. Morpholino oligonucleotide knockdown of *Toe1* caused midbrain and hindbrain degeneration.[50] TOE1 has roles in RNA degradation, and variants in *TOE1* have been reported in affected individuals with PCH7. TSEN variants and the associated protein CLP1 have been associated with PCH in affected individuals.[51,52] Morpholino knockdown of these genes in zebrafish embryos also recapitulated the phenotype seen in affected individuals. Together, these studies highlight the consistency of zebrafish as a model of cerebellar hypoplasia and atrophy in particular in disorders of RNA processing.

Morpholino knockdown of *exosc9* has previously been performed in *Xenopus* embryos.[53] However, the *Xenopus exosc9* morphants had defects in skin development but not the cerebellum. The discrepancy between zebrafish and *Xenopus* could be due to inherent differences in the models, or it could simply be that the cerebellum was not examined in the *Xenopus* morphants. Our results here show that zebrafish are a useful tool for generating rapid *in vivo* models of rare genetic diseases involving the development of the brain and spinal cord.

In summary, we have described four independent individuals who are affected by variants in *EXOSC9* and who presented with motor axonopathy resembling SMA, cerebellar atrophy, and in one affected individual, multiple bone fractures. These *EXOSC9*-related phenotypes closely resemble the clinical spectrum of other exosomal defects, which could be referred to as "exosomopathies." The clinically unique combination of a motor neuronopathy with cerebellar atrophy typical for the exosomopathies link the other two major clinical groups of the disorders of RNA processing, namely SMA without cerebellar involvement on the one end and the pontocerebellar hypoplasias without SMA on the other. Molecular studies indicate that the pathology is linked to a loss of function of the RNA processing by the exosome. Further studies on neuronal cells of affected individuals or zebrafish neurons might reveal neuron-specific alterations, which could be targeted in future interventions.

Accession Numbers

We have deposited WES and phenotypic data of affected individual 2:II-1 into the European Genome-phenome Archive (EGA: EGAN00001679530). WES data of affected individual 1:II-1 are scheduled to be deposited to dbGaP. The genetic data of affected individual 4:II-1 have been submitted to ClinVar.

Supplemental Data

Supplemental Data include one figure, four tables and one video and can be found with this article online at https://doi.org/10.1016/j.ajhg.2018.03.011.

Acknowledgments

We thank the families for participating and Christopher Mendoza and Gilberto ("Mike") Averion for their help in the clinic. We would also like to thank the NIH Intramural Sequencing Center staff and Daniel MacArthur and Fengmei Zhao (Analytic and Translational Genetics Unit at Massachusetts General Hospital in collaboration with the Broad Institute of Harvard and Massachusetts Institute of Technology) for their help with exome analysis. We also thank the Exome Aggregation Consortium and the groups that provided exome variant data for comparison. A full list of contributing groups can be found at http://exac.broadinstitute.org/about. R.H. is a Wellcome Investigator (109915/Z/15/Z) who receives support from the Wellcome Centre for Mitochondrial Research (203105/Z/16/Z), Medical Research Council (UK) (MR/N025431/1), the European Research Council (309548), the

Wellcome Trust Pathfinder Scheme (201064/Z/16/Z), and the Newton Fund (UK/Turkey, MR/N027302/1). Work in C.G. Bönnemann's laboratory is supported by intramural funds from the NIH National Institute of Neurological Disorders and Stroke. Exome sequencing was funded through the Clinical Center Genomics Opportunity, which is sponsored by the National Human Genome Research Institute, the NIH Deputy Director for Intramural Research, and the NIH Clinical Center. Sequencing analysis was provided by the Broad Institute of MIT and Harvard Center for Mendelian Genomics and was funded by the National Human Genome Research Institute, the National Eye Institute, and National Heart, Lung, and Blood Institute grant UM1 HG008900 to Daniel MacArthur and Heidi Rehm. Work in M.S.'s laboratory was supported by the Deutsche Forschungsgemeinschaft (grants SFB 665 TP C4 and NeuroCure Exc 257).

Received: December 15, 2017
Accepted: March 6, 2018
Published: May 3, 2018

Web Resources

1000 Genomes Project, http://www.internationalgenome.org/
ClinVar, https://www.ncbi.nlm.nih.gov/clinvar/
ConsensusPathDB (CPDB), http://cpdb.molgen.mpg.de/
dbGaP, http://www.ncbi.nlm.nih.gov/gap
dbSNP, https://www.ncbi.nlm.nih.gov/SNP/
European Genome-phenome Archive (EGA), https://www.ebi.ac.uk/ega/home
ExAC Browser, http://exac.broadinstitute.org/
FastQC, http://www.bioinformatics.babraham.ac.uk/projects/fastqc/
GATK, https://software.broadinstitute.org/gatk/
GenBank, https://www.ncbi.nlm.nih.gov/genbank/
GeneDx ClinVar, http://www.ncbi.nlm.nih.gov/clinvar/submitters/26957/
IGV, http://www.broadinstitute.org/igv/
OMIM, https://www.omim.org/
UCSC Genome Browser, https://genome.ucsc.edu/

References

1. Lehner, B., and Sanderson, C.M. (2004). A protein interaction framework for human mRNA degradation. Genome Res. *14*, 1315–1323.
2. Estévez, A.M., Lehner, B., Sanderson, C.M., Ruppert, T., and Clayton, C. (2003). The roles of intersubunit interactions in exosome stability. J. Biol. Chem. *278*, 34943–34951.
3. Allmang, C., Petfalski, E., Podtelejnikov, A., Mann, M., Tollervey, D., and Mitchell, P. (1999). The yeast exosome and human PM-Scl are related complexes of 3′–> 5′ exonucleases. Genes Dev. *13*, 2148–2158.
4. Makino, D.L., Baumgärtner, M., and Conti, E. (2013). Crystal structure of an RNA-bound 11-subunit eukaryotic exosome complex. Nature *495*, 70–75.
5. Staals, R.H.J., and Pruijn, G.J.M. (2010). The human exosome and disease. In RNA Exosome. Advances in Experimental Medicine and Biology, *vol 702*, Jensen T.H., ed. *(Springer), pp. 132–142*.
6. Allmang, C., Kufel, J., Chanfreau, G., Mitchell, P., Petfalski, E., and Tollervey, D. (1999). Functions of the exosome in rRNA, snoRNA and snRNA synthesis. EMBO J. *18*, 5399–5410.
7. Kadaba, S., Krueger, A., Trice, T., Krecic, A.M., Hinnebusch, A.G., and Anderson, J. (2004). Nuclear surveillance and degradation of hypomodified initiator tRNAMet in S. cerevisiae. Genes Dev. *18*, 1227–1240.
8. Wyers, F., Rougemaille, M., Badis, G., Rousselle, J.-C., Dufour, M.-E., Boulay, J., Régnault, B., Devaux, F., Namane, A., Séraphin, B., et al. (2005). Cryptic pol II transcripts are degraded by a nuclear quality control pathway involving a new poly(A) polymerase. Cell *121*, 725–737.
9. Gudipati, R.K., Xu, Z., Lebreton, A., Séraphin, B., Steinmetz, L.M., Jacquier, A., and Libri, D. (2012). Extensive degradation of RNA precursors by the exosome in wild-type cells. Mol. Cell *48*, 409–421.
10. Bousquet-Antonelli, C., Presutti, C., and Tollervey, D. (2000). Identification of a regulated pathway for nuclear pre-mRNA turnover. Cell *102*, 765–775.
11. Sayani, S., and Chanfreau, G.F. (2012). Sequential RNA degradation pathways provide a fail-safe mechanism to limit the accumulation of unspliced transcripts in Saccharomyces cerevisiae. RNA *18*, 1563–1572.
12. van Hoof, A., Lennertz, P., and Parker, R. (2000). Yeast exosome mutants accumulate 3′-extended polyadenylated forms of U4 small nuclear RNA and small nucleolar RNAs. Mol. Cell. Biol. *20*, 441–452.
13. Büttner, K., Wenig, K., and Hopfner, K.P. (2006). The exosome: a macromolecular cage for controlled RNA degradation. Mol. Microbiol. *61*, 1372–1379.
14. Lubas, M., Christensen, M.S., Kristiansen, M.S., Domanski, M., Falkenby, L.G., Lykke-Andersen, S., Andersen, J.S., Dziembowski, A., and Jensen, T.H. (2011). Interaction profiling identifies the human nuclear exosome targeting complex. Mol. Cell *43*, 624–637.
15. Hrossova, D., Sikorsky, T., Potesil, D., Bartosovic, M., Pasulka, J., Zdrahal, Z., Stefl, R., and Vanacova, S. (2015). RBM7 subunit of the NEXT complex binds U-rich sequences and targets 3′-end extended forms of snRNAs. Nucleic Acids Res. *43*, 4236–4248.
16. Wan, J., Yourshaw, M., Mamsa, H., Rudnik-Schöneborn, S., Menezes, M.P., Hong, J.E., Leong, D.W., Senderek, J., Salman, M.S., Chitayat, D., et al. (2012). Mutations in the RNA exosome component gene EXOSC3 cause pontocerebellar hypoplasia and spinal motor neuron degeneration. Nat. Genet. *44*, 704–708.
17. Boczonadi, V., Müller, J.S., Pyle, A., Munkley, J., Dor, T., Quartararo, J., Ferrero, I., Karcagi, V., Giunta, M., Polvikoski, T., et al. (2014). EXOSC8 mutations alter mRNA metabolism and cause hypomyelination with spinal muscular atrophy and cerebellar hypoplasia. Nat. Commun. *5*, 4287.
18. Rudnik-Schöneborn, S., Senderek, J., Jen, J.C., Houge, G., Seeman, P., Puchmajerová, A., Graul-Neumann, L., Seidel, U., Korinthenberg, R., Kirschner, J., et al. (2013). Pontocerebellar hypoplasia type 1: clinical spectrum and relevance of EXOSC3 mutations. Neurology *80*, 438–446.
19. Giunta, M., Edvardson, S., Xu, Y., Schuelke, M., Gomez-Duran, A., Boczonadi, V., Elpeleg, O., Müller, J.S., and Horvath, R. (2016). Altered RNA metabolism due to a homozygous RBM7 mutation in a patient with spinal motor neuropathy. Hum. Mol. Genet. *25*, 2985–2996.
20. Sobreira, N., Schiettecatte, F., Boehm, C., Valle, D., and Hamosh, A. (2015). New tools for Mendelian disease

gene identification: PhenoDB variant analysis module; and GeneMatcher, a web-based tool for linking investigators with an interest in the same gene. Hum. Mutat. *36*, 425–431.
21. Lek, M., Karczewski, K.J., Minikel, E.V., Samocha, K.E., Banks, E., Fennell, T., O'Donnell-Luria, A.H., Ware, J.S., Hill, A.J., Cummings, B.B., et al.; Exome Aggregation Consortium (2016). Analysis of protein-coding genetic variation in 60,706 humans. Nature *536*, 285–291.
22. Teer, J.K., Green, E.D., Mullikin, J.C., and Biesecker, L.G. (2012). VarSifter: visualizing and analyzing exome-scale sequence variation data on a desktop computer. Bioinformatics *28*, 599–600.
23. Li, M.-X., Kwan, J.S.H., Bao, S.-Y., Yang, W., Ho, S.-L., Song, Y.-Q., and Sham, P.C. (2013). Predicting mendelian disease-causing non-synonymous single nucleotide variants in exome sequencing studies. PLoS Genet. *9*, e1003143.
24. DePristo, M.A., Banks, E., Poplin, R., Garimella, K.V., Maguire, J.R., Hartl, C., Philippakis, A.A., del Angel, G., Rivas, M.A., Hanna, M., et al. (2011). A framework for variation discovery and genotyping using next-generation DNA sequencing data. Nat. Genet. *43*, 491–498.
25. Schwarz, J.M., Cooper, D.N., Schuelke, M., and Seelow, D. (2014). MutationTaster2: mutation prediction for the deep-sequencing age. Nat. Methods *11*, 361–362.
26. Group, S.M.; and Saudi Mendeliome Group (2015). Comprehensive gene panels provide advantages over clinical exome sequencing for Mendelian diseases. Genome Biol. *16*, 134.
27. Tanaka, A.J., Cho, M.T., Millan, F., Juusola, J., Retterer, K., Joshi, C., Niyazov, D., Garnica, A., Gratz, E., Deardorff, M., et al. (2015). Mutations in SPATA5 Are Associated with Microcephaly, Intellectual Disability, Seizures, and Hearing Loss. Am. J. Hum. Genet. *97*, 457–464.
28. Lake, N.J., Webb, B.D., Stroud, D.A., Richman, T.R., Ruzzenente, B., Compton, A.G., Mountford, H.S., Pulman, J., Zangarelli, C., Rio, M., et al. (2017). Biallelic Mutations in MRPS34 Lead to Instability of the Small Mitoribosomal Subunit and Leigh Syndrome. Am. J. Hum. Genet. *101*, 239–254.
29. Schwabova, J., Brozkova, D.S., Petrak, B., Mojzisova, M., Pavlickova, K., Haberlova, J., Mrazkova, L., Hedvicakova, P., Hornofova, L., Kaluzova, M., et al. (2013). Homozygous EXOSC3 mutation c.92G→C, p.G31A is a founder mutation causing severe pontocerebellar hypoplasia type 1 among the Czech Roma. J. Neurogenet. *27*, 163–169.
30. Fasken, M.B., Losh, J.S., Leung, S.W., Brutus, S., Avin, B., Vaught, J.C., Potter-Birriel, J., Craig, T., Conn, G.L., Mills-Lujan, K., et al. (2017). Insight into the RNA Exosome Complex Through Modeling Pontocerebellar Hypoplasia Type 1b Disease Mutations in Yeast. Genetics *205*, 221–237.
31. Drmanac, R., Sparks, A.B., Callow, M.J., Halpern, A.L., Burns, N.L., Kermani, B.G., Carnevali, P., Nazarenko, I., Nilsen, G.B., Yeung, G., et al. (2010). Human genome sequencing using unchained base reads on self-assembling DNA nanoarrays. Science *327*, 78–81.
32. Schottmann, G., Picker-Minh, S., Schwarz, J.M., Gill, E., Rodenburg, R.J.T., Stenzel, W., Kaindl, A.M., and Schuelke, M. (2017). Recessive mutation in EXOSC3 associates with mitochondrial dysfunction and pontocerebellar hypoplasia. Mitochondrion *37*, 46–54.
33. Anders, S., Pyl, P.T., and Huber, W. (2015). HTSeq–a Python framework to work with high-throughput sequencing data. Bioinformatics *31*, 166–169.
34. Love, M.I., Huber, W., and Anders, S. (2014). Moderated estimation of fold change and dispersion for RNA-seq data with DESeq2. Genome Biol. *15*, 550.
35. Westerfield, M. (1993). The zebrafish book: a guide for the laboratory use of zebrafish (Brachydanio rerio) (Inst. of Neuroscience, University of Oregon).
36. Moreno-Mateos, M.A., Vejnar, C.E., Beaudoin, J.-D., Fernandez, J.P., Mis, E.K., Khokha, M.K., and Giraldez, A.J. (2015). CRISPRscan: designing highly efficient sgRNAs for CRISPR-Cas9 targeting in vivo. Nat. Methods *12*, 982–988.
37. Varshney, G.K., Pei, W., LaFave, M.C., Idol, J., Xu, L., Gallardo, V., Carrington, B., Bishop, K., Jones, M., Li, M., et al. (2015). High-throughput gene targeting and phenotyping in zebrafish using CRISPR/Cas9. Genome Res. *25*, 1030–1042.
38. Varshney, G.K., Carrington, B., Pei, W., Bishop, K., Chen, Z., Fan, C., Xu, L., Jones, M., LaFave, M.C., Ledin, J., et al. (2016). A high-throughput functional genomics workflow based on CRISPR/Cas9-mediated targeted mutagenesis in zebrafish. Nat. Protoc. *11*, 2357–2375.
39. Kimmel, C.B., Ballard, W.W., Kimmel, S.R., Ullmann, B., and Schilling, T.F. (1995). Stages of embryonic development of the zebrafish. Dev. Dyn. *203*, 253–310.
40. Meeker, N.D., Hutchinson, S.A., Ho, L., and Trede, N.S. (2007). Method for isolation of PCR-ready genomic DNA from zebrafish tissues. Biotechniques *43*, 610–, 612, 614.
41. Arnold, K., Bordoli, L., Kopp, J., and Schwede, T. (2006). The SWISS-MODEL workspace: a web-based environment for protein structure homology modelling. Bioinformatics *22*, 195–201.
42. Ying, D., Sham, P.C., Smith, D.K., Zhang, L., Lau, Y.L., and Yang, W. (2015). HaploShare: identification of extended haplotypes shared by cases and evaluation against controls. Genome Biol. *16*, 92.
43. Higashijima, S., Hotta, Y., and Okamoto, H. (2000). Visualization of cranial motor neurons in live transgenic zebrafish expressing green fluorescent protein under the control of the islet-1 promoter/enhancer. J. Neurosci. *20*, 206–218.
44. McWhorter, M.L., Monani, U.R., Burghes, A.H.M., and Beattie, C.E. (2003). Knockdown of the survival motor neuron (Smn) protein in zebrafish causes defects in motor axon outgrowth and pathfinding. J. Cell Biol. *162*, 919–931.
45. Knierim, E., Hirata, H., Wolf, N.I., Morales-Gonzalez, S., Schottmann, G., Tanaka, Y., Rudnik-Schöneborn, S., Orgeur, M., Zerres, K., Vogt, S., et al. (2016). Mutations in Subunits of the Activating Signal Cointegrator 1 Complex Are Associated with Prenatal Spinal Muscular Atrophy and Congenital Bone Fractures. Am. J. Hum. Genet. *98*, 473–489.
46. Namavar, Y., Barth, P.G., Poll-The, B.T., and Baas, F. (2011). Classification, diagnosis and potential mechanisms in pontocerebellar hypoplasia. Orphanet J. Rare Dis. *6*, 50.
47. Eggens, V.R.C., Barth, P.G., Niermeijer, J.-M.F., Berg, J.N., Darin, N., Dixit, A., Fluss, J., Foulds, N., Fowler, D., Hortobágyi, T., et al. (2014). EXOSC3 mutations in pontocerebellar hypoplasia type 1: novel mutations and genotype-phenotype correlations. Orphanet J. Rare Dis. *9*, 23.
48. Kok, F.O., Shin, M., Ni, C.W., Gupta, A., Grosse, A.S., van Impel, A., Kirchmaier, B.C., Peterson-Maduro, J., Kourkoulis, G., Male, I., et al. (2015). Reverse genetic screening reveals poor correlation between morpholino-induced and mutant phenotypes in zebrafish. Dev. Cell *32*, 97–108.

49. Sharma, K.R., Heckler, K., Stoll, S.J., Hillebrands, J.-L., Kynast, K., Herpel, E., Porubsky, S., Elger, M., Hadaschik, B., Bieback, K., et al. (2016). ELMO1 protects renal structure and ultrafiltration in kidney development and under diabetic conditions. Sci. Rep. *6*, 37172.

50. Lardelli, R.M., Schaffer, A.E., Eggens, V.R.C., Zaki, M.S., Grainger, S., Sathe, S., Van Nostrand, E.L., Schlachetzki, Z., Rosti, B., Akizu, N., et al. (2017). Biallelic mutations in the 3′ exonuclease TOE1 cause pontocerebellar hypoplasia and uncover a role in snRNA processing. Nat. Genet. *49*, 457–464.

51. Kasher, P.R., Namavar, Y., van Tijn, P., Fluiter, K., Sizarov, A., Kamermans, M., Grierson, A.J., Zivkovic, D., and Baas, F. (2011). Impairment of the tRNA-splicing endonuclease subunit 54 (tsen54) gene causes neurological abnormalities and larval death in zebrafish models of pontocerebellar hypoplasia. Hum. Mol. Genet. *20*, 1574–1584.

52. Schaffer, A.E., Eggens, V.R., Caglayan, A.O., Reuter, M.S., Scott, E., Coufal, N.G., Silhavy, J.L., Xue, Y., Kayserili, H., Yasuno, K., et al. (2014). CLP1 founder mutation links tRNA splicing and maturation to cerebellar development and neurodegeneration. Cell *157*, 651–663.

53. Noiret, M., Mottier, S., Angrand, G., Gautier-Courteille, C., Lerivray, H., Viet, J., Paillard, L., Mereau, A., Hardy, S., and Audic, Y. (2016). Ptbp1 and Exosc9 knockdowns trigger skin stability defects through different pathways. Dev. Biol. *409*, 489–501.

The American Journal of Human Genetics, Volume *102*

Supplemental Data

Variants in *EXOSC9* Disrupt the RNA Exosome and Result in Cerebellar Atrophy with Spinal Motor Neuronopathy

David T. Burns, Sandra Donkervoort, Juliane S. Müller, Ellen Knierim, Diana Bharucha-Goebel, Eissa Ali Faqeih, Stephanie K. Bell, Abdullah Y. AlFaifi, Dorota Monies, Francisca Millan, Kyle Retterer, Sarah Dyack, Sara MacKay, Susanne Morales-Gonzalez, Michele Giunta, Benjamin Munro, Gavin Hudson, Mena Scavina, Laura Baker, Tara C. Massini, Monkol Lek, Ying Hu, Daniel Ezzo, Fowzan S. AlKuraya, Peter B. Kang, Helen Griffin, A. Reghan Foley, Markus Schuelke, Rita Horvath, and Carsten G. Bönnemann

Figure S1:

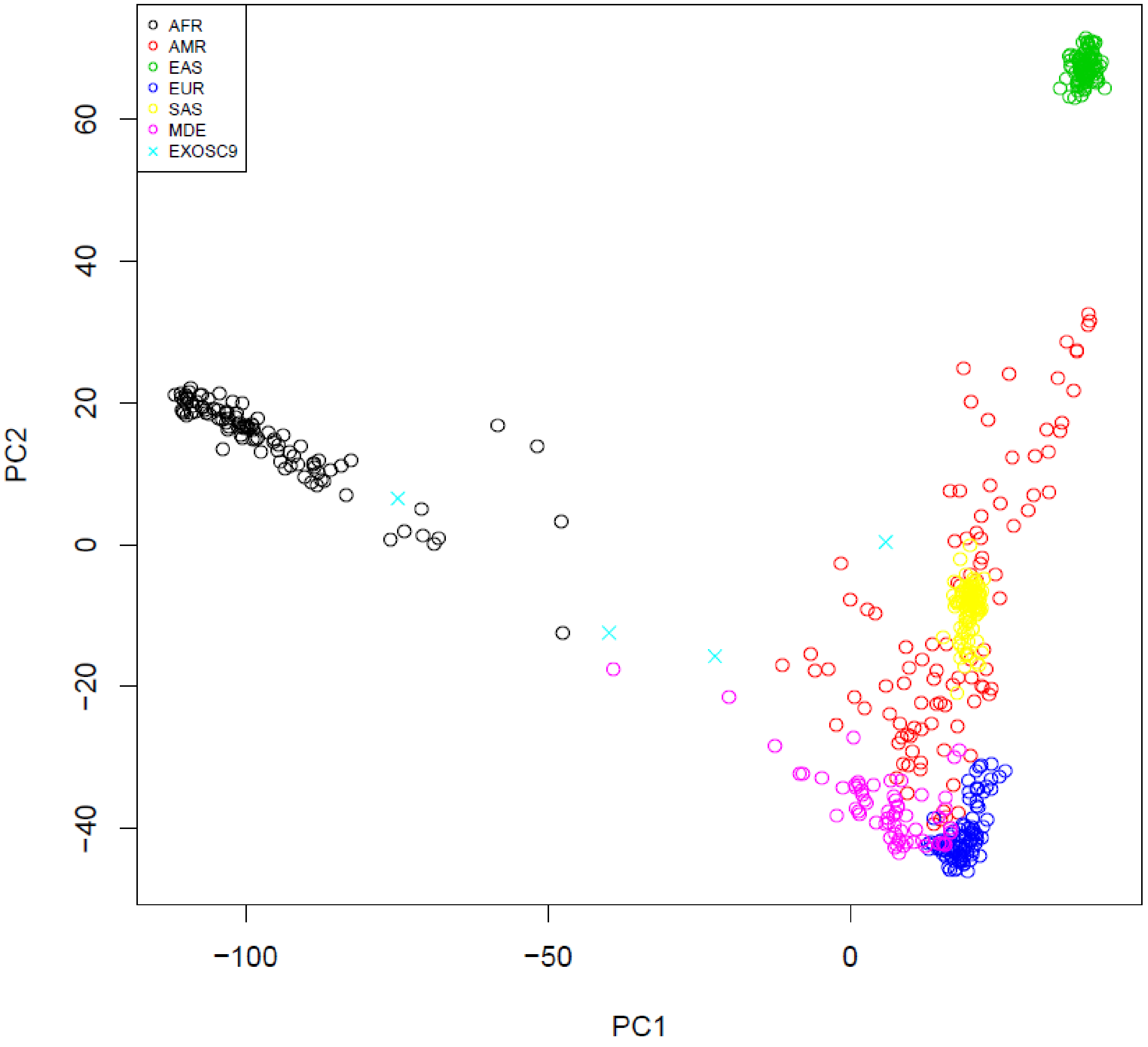

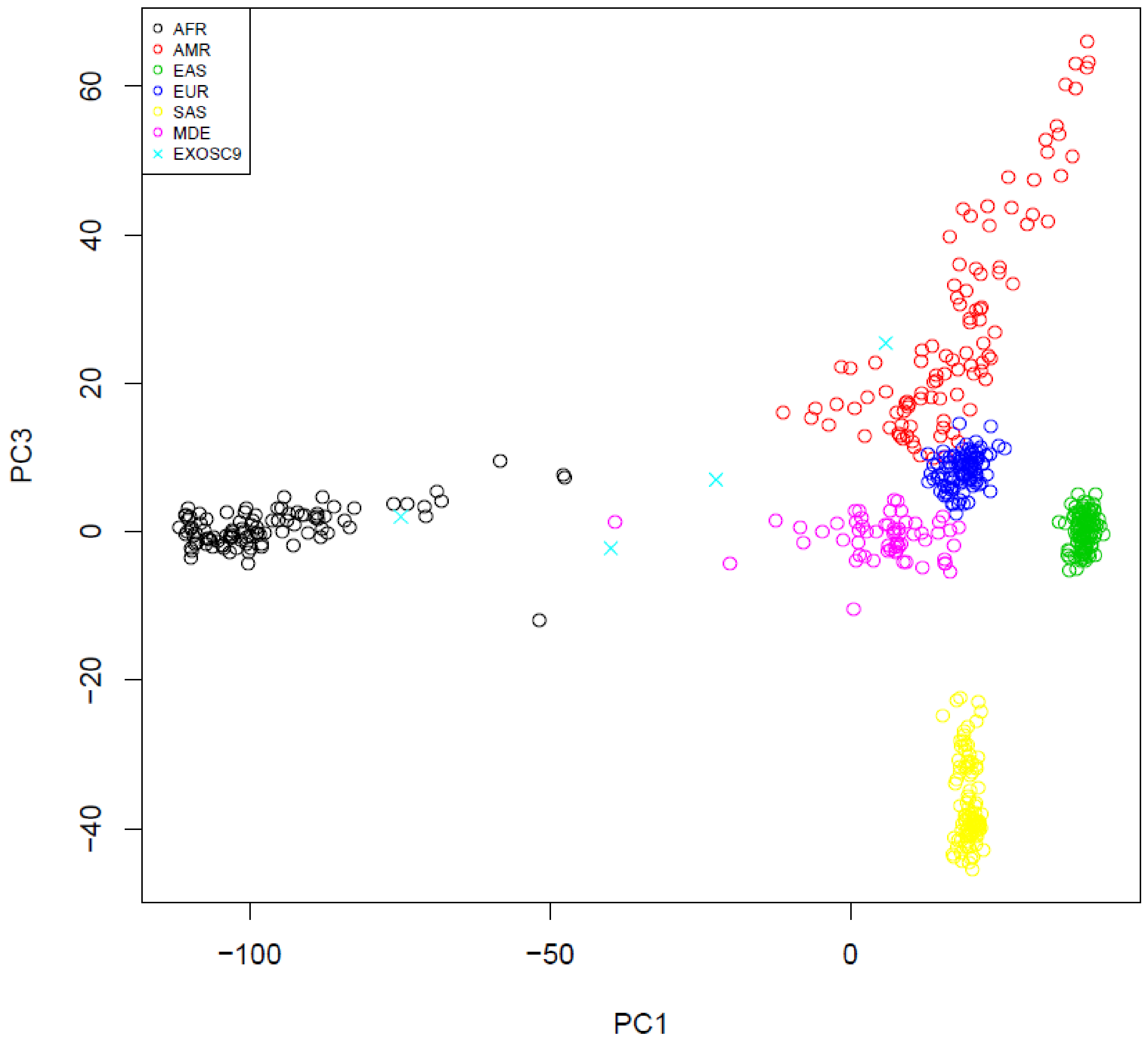
AFR
AMR
EAS
EUR
SAS
MDE
EXOSC9
PC3
PC1
60
40
20
0
-20
-40
-100
-50
0

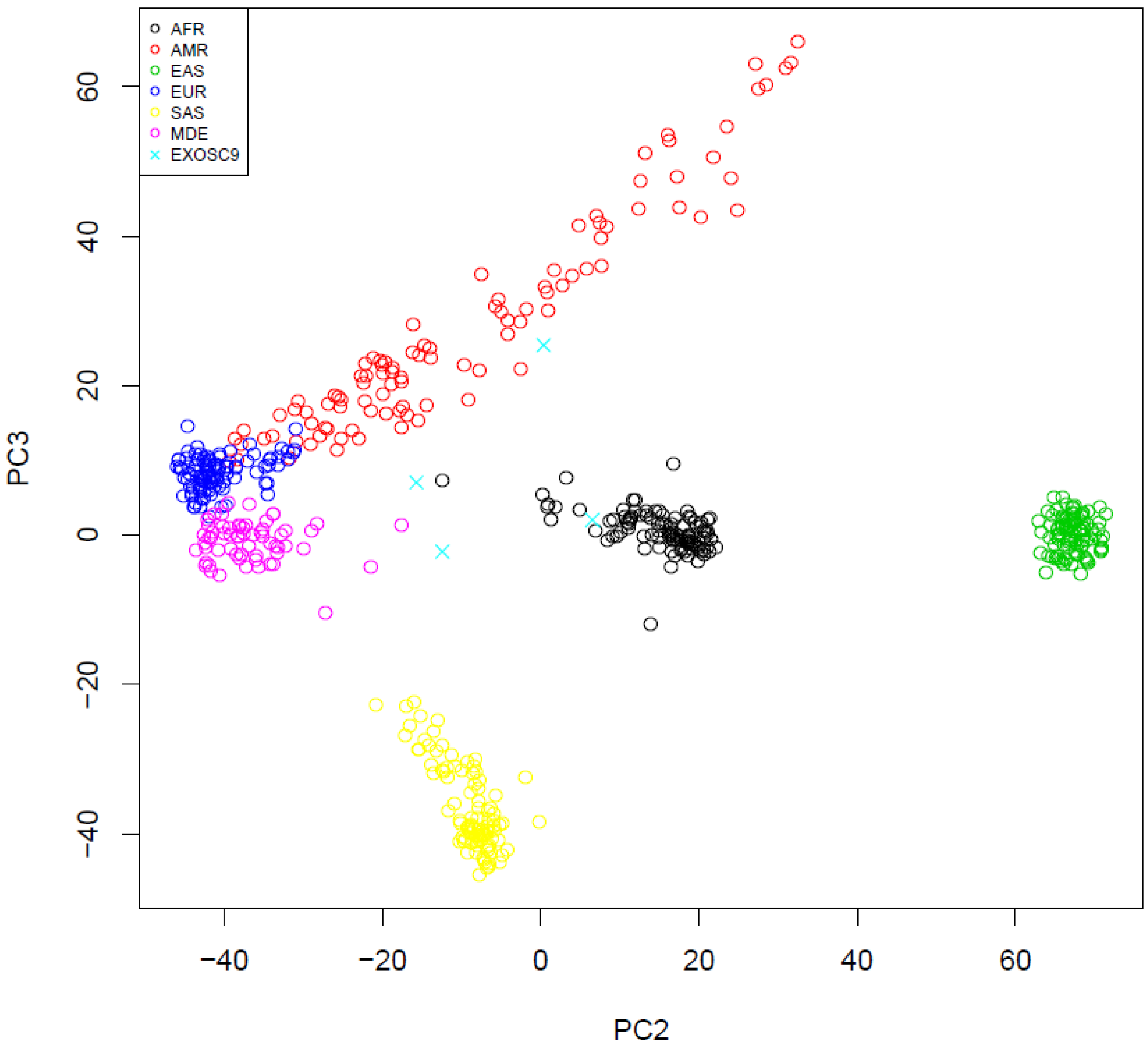

AFR
AMR
EAS
EUR
SAS
MDE
EXOSC9
PC3
PC2
60
40
20
0
−20
−40
−40
−20
0
20
40
60

Figure S1: Principal Component Analysis of exome SNP genotypes compared against random representative samples from the 1000 Genomes Projects Phase 3 super-populations plus a GeneDx-sequenced Middle Eastern (MDE) population. Five total components were calculated (see Table) but only the three most informative components are shown.

Table S1:

Primer name	Forward 3´-5´	Reverse 3´-5´
ZF *exosc9* (I1E2 MO)	CAAACTGCGAGAGGCTTTTCC	TCTCCTGAAACTACACAGAGAG
ZF *exosc9* (E3 gRNA)	TGAGTCAGGTATCCTGTGAGC	TGCAAGAATGTTAACCTGTTTGGTT
pUC/M13	CGCCAGGGTTTTCCCAGTCACGAC	TCACACAGGAAACAGCTATGAC
**Morpholino**	**Sequence**	
ZF *exosc9* morpholino	GCGCTACAAAACGGTACACAGATAA	

Table S1: Primer sequences used in zebrafish

Table S2:

	Characteristics and Symptoms	HPO ID	Individual 1	Individual 2	Individual 3	Individual 4	Percentage
Mutation in EXOSC9			homo c.41T>C	comp het c.41T>C | c.481C>T	homo c.41T>C	homo c.41T>C	
Effect on translation			p.(Leu14Pro)	p.(Leu14Pro), p.(Arg161*)	p.(Leu14Pro)	p.(Leu14Pro)	
Origin			El Salvador	African-Canadian-Jamaican	Saudi Arabia	African-European-Filippino	
Gender			female	male	female	female	
Age at onset			8 mo	birth	birth	2 weeks	
Age at last assessment			28 mo		4.5 y	19 mo	
Age at death				15 mo			
**Head**							
	Microcephalus	HP:0000252			+		25
	Low-set ears	HP:0000369		+			25
	Short neck	HP:0000470		+			25
	Blue sclerae	HP:0000592		+			25
	Prominent epicanthic folds	HP:0007930		+			25
	Hypertelorism	HP:0000316		+			25
**Respiratory and chest**							
	Recurrent infections	HP:0002719	+				25
	Respiratory failure in infancy	HP:0002878	+	+			50
**Abdomen**							
	Gastrostomy tube feeding in infancy	HP:0011471	+	+			50
	Gastrooesophageal reflux	HP:0002020	+				25
	Feeding difficulties in infancy	HP:0008872			+		25
**Skeletal**							
	Arthrogryposis multiplex congenital	HP:0002804	+	+			50
	Congenital fractures	HP:0005855		+			25
**Neurologic**							
	Severe muscular hypotonia	HP:0006829	+	+	+	+	**100**
	Seizures	HP:0001250			+		25
	Type I muscle fiber predominance	HP:0003803	+	+		+	**75**
	Poor head control	HP:0002421	+			+	50
	Failure to thrive	HP:0001508		+			25
	Generalized muscle weakness	HP:0003324	+		+	+	**75**
	Nystagmus	HP: 0000639	+			+	50
	Cerebellar atrophy	HP:0001272	+	+		+	75
	Vermis atrophy	HP:0001284	+				25
	Cerebral atrophy	HP:0002059		+	+		50
	Areflexia	HP:0001284	+				25
	Increased muscle reflexes	HP:0001347			+		25
	EMG: neuropathic changes	HP:0003445	+			+	50
	Muscle / tongue fasciculations	HP:0002380	+				25
	Delayed gross motor development	HP:0002194	+	+	+	+	**100**
	Weak voice	HP:0001621	+		+	+	**75**
**Prenatal manifestation**							
	Decreased fetal movements	HP:0001558	+	+			30
	Oligohydramnios	HP:0001562		+			25
	Intrauterine growth retardation (< 10th percentile)	HP:0001511		+			25

Table S2: HPO coding of phenotypes due to mutations in *EXOSC9*

Table S3:

Sample	knn.pred	PC1	PC2	PC3	PC4	PC5
**Individual 1**	AMR	5.86084161991711	0.381779639737347	25.4032514365166	-21.0875664424482	18.6590532074565
**Individual 2**	AMR	-22.3702289478425	-15.7062479478535	7.02632626688231	13.8203817951703	34.1568567434397
**Individual 3**	AFR	-39.8713917778595	-12.421418841825	-2.22439179095596	17.719501768131	39.8300579056514
**Individual 4**	AFR	-74.7967964546837	6.5332515180803	2.02902721367773	3.38496964411094	3.05927032223124

Table S3: Principal Component Analysis of exome SNP genotypes compared against random representative samples from the 1000 Genomes Projects Phase 3 major populations plus a GeneDx-sequenced Middle Eastern (MDE) population. Components are numbered in the order of informativeness and knn.pred is the nearest-neighbor-predicted population.
